# Migration of Lung Resident Group 2 Innate Lymphoid Cells Link Allergic Lung Inflammation and Liver Immunity

**DOI:** 10.3389/fimmu.2021.679509

**Published:** 2021-07-09

**Authors:** Laura Mathä, Mónica Romera-Hernández, Catherine A. Steer, Yi Han Yin, Mona Orangi, Hanjoo Shim, ChihKai Chang, Fabio M. Rossi, Fumio Takei

**Affiliations:** ^1^ Terry Fox Laboratory, British Columbia Cancer, Vancouver, BC, Canada; ^2^ Interdisciplinary Oncology Program, University of British Columbia, Vancouver, BC, Canada; ^3^ Department of Pathology and Laboratory Medicine, University of British Columbia, Vancouver, BC, Canada; ^4^ Biomedical Research Centre, University of British Columbia, Vancouver, BC, Canada; ^5^ Department of Medical Genetics, University of British Columbia, Vancouver, BC, Canada

**Keywords:** group 2 innate lymphoid cells, allergic lung inflammation, tissue resident, migration, liver inflammation, parabiosis

## Abstract

Group 2 innate lymphoid cells (ILC2s) are tissue resident in the lung and activated by inhaled allergens *via* epithelial-derived alarmins including IL-33. Activated ILC2s proliferate, produce IL-5 and IL-13, and induce eosinophilic inflammation. Here, we report that intranasal IL-33 or the protease allergen papain administration resulted in increased numbers of ILC2s not only in the lung but also in peripheral blood and liver. Analyses of IL-33 treated parabiosis mice showed that the increase in lung ILC2s was due to proliferation of lung resident ILC2s, whereas the increase in liver ILC2s was due to the migration of activated lung ILC2s. Lung-derived ILC2s induced eosinophilic hepatitis and expression of fibrosis-related genes. Intranasal IL-33 pre-treatment also attenuated concanavalin A-induced acute hepatitis and cirrhosis. These results suggest that activated lung resident ILC2s emigrate from the lung, circulate, settle in the liver and promote type 2 inflammation and attenuate type 1 inflammation.

## Introduction

Group 2 innate lymphoid cells (ILC2s) have been identified in mucosal and non-mucosal tissues, including the gut (1–3), lung (4), skin (5), adipose tissues (6, 7) and the liver (8). They do not express T, B or myeloid cell lineage markers (Lin^-^), but express IL-7R (CD127) and CD90 (Thy1) (1–3). They highly express the transcription factors GATA3 (1, 9, 10) and RAR-related orphan receptor alpha (RORα) (4), and ILC2s are deficient in *Rora* mutant Staggerer mice (11, 12). ILC2s in the lung express IL-33R (T1/ST2), the receptor for IL-33, and are activated by inhaled allergens that induce IL-33 release from airway epithelial cells. Activated lung ILC2s produce copious amounts of type 2 cytokines IL-5 and IL-13 (13), which induce eosinophilia (14), mucus hyperproduction (15) and airway hyperresponsiveness (16).

ILC2s have been implicated in various diseases including asthma and liver fibrosis (17). In mice, ILC2s have been demonstrated to play key roles in papain (4), house dust mite (4, 18), ovalbumin (18, 19), *Aspergillus oryzae* (20), and *Alternaria alternata* (21, 22) models of asthma. Likewise in humans, elevated numbers of ILC2s are found in the peripheral blood (PB) (23) and bronchoalveolar lavage fluid (24) of asthmatic patients, suggesting a similar role in human disease. In the liver, the number of ILC2s increases proportionally to the severity of fibrotic disease (25), while IL-33, a potent stimulator of ILC2s, is elevated in the serum of liver cirrhosis patients (8). In mouse models, ILC2s are involved in the development of hepatic fibrosis (8) and concanavalin A (ConA)-induced immune mediated hepatitis (26), while activated ILC2s have been shown to exert mild protective effects in adenovirus-induced viral hepatitis (27).

ILC2s have previously been shown to be tissue resident at steady state, as well as during systemic autoimmunity and parasitic infections (28, 29). However, recent reports have suggested that upon activation, ILC2s leave the bone marrow (BM) and may be recruited to sites of inflammation (30, 31). Stier et al. intravenously (i.v.) administered IL-33 into mice and found egress of ILC2 progenitors from the BM, while Karta et al. demonstrated that ILC2s (Lin^-^Thy1^+^ cells) were recruited from the BM to the lung in mice receiving intranasal (i.n.) injections of *Alternaria alternata*. They also showed that this process was mediated by β2 integrins. Huang et al. reported that intraperitoneal (i.p.) injections of IL-25 induced migration of KLRG1^+^ inflammatory ILC2s from the intestine to the lung and differentiation into “natural” ILC2-like cells (32, 33). This was through sphingosine 1-phosphate (S1P) mediated chemotaxis (33). More recently, Ricardo-Gonzalez et al. described the ability of intestinal and lung ILC2s to circulate upon *Nippostrongylus brasiliensis* infection, demonstrating that ILC2s can mediate transition of local immune response to systemic type 2 responses (34). We have previously shown that i.n. administrations of the protease allergen papain induce IL-33-dependent activation of lung ILC2s and increases in ILC2 numbers not only in the lung but also in the draining mediastinal lymph node (LN) (20), suggesting that activated lung ILC2s might migrate out of the lung and enter the lymphatic system.

Here, we report that i.n. administrations of papain or IL-33 induced an increase in ILC2 numbers not only in the lung but also in PB and liver. Our analyses of IL-33 treated parabiotic mice indicated that a subset of activated lung resident ILC2s emigrates from the lung, circulates and preferentially settles in the liver. Further analyses of the lung-derived ILC2s in the liver showed two apparently opposing effects, namely promoting eosinophilic hepatitis and attenuating ConA-induced acute hepatitis and cirrhosis. In summary, migration of lung ILC2s links allergic lung inflammation and liver immunity and inflammation.

## Materials and Methods

### Mice

C57BL/6J (B6), B6.SJL-*Ptprc* ^a^ *Pepc* ^b^/BoyJ (Pep3b) and B6.129P2(Cg)-*Rorc* ^tm2Litt^/J (*Rorc* ^-/-^) mice were bred in the British Columbia Cancer Research Centre (BCCRC) and Biomedical Research Centre (BRC) animal facilities from breeder mice purchased from the Jackson Laboratory (stock #: 000664, 002014, 007572, respectively), and CD127 cKO mice were generated in house. Animals were maintained in the BCCRC or BRC (parabiosis mice) specific pathogen-free animal facility. C57BL/6-*Itgb7^tm1Cgn^*/J (*Itgb7* ^-/-^), B6.129S2-*Il6^tm1Kopf^*/J (*Il6* ^-/-^) (35) and B6.129P2-*Cxcr6^tm1Litt^*/J (*Cxcr6* ^gfp/gfp^) mice were purchased from the Jackson Laboratory (stock #: 002965, 002650, 005693, respectively). All animal use was approved by the animal care committee of the University of British Columbia and were maintained and euthanized in accordance with the guidelines of the Canadian Council on Animal Care. Briefly, mice were housed in vented or static cages (4 mice maximum per cage) with crinkle paper nesting materials and a hiding place. Parabiosis mouse pairs were housed singly in static cages placed on a heat pad.

**Figure 1:**
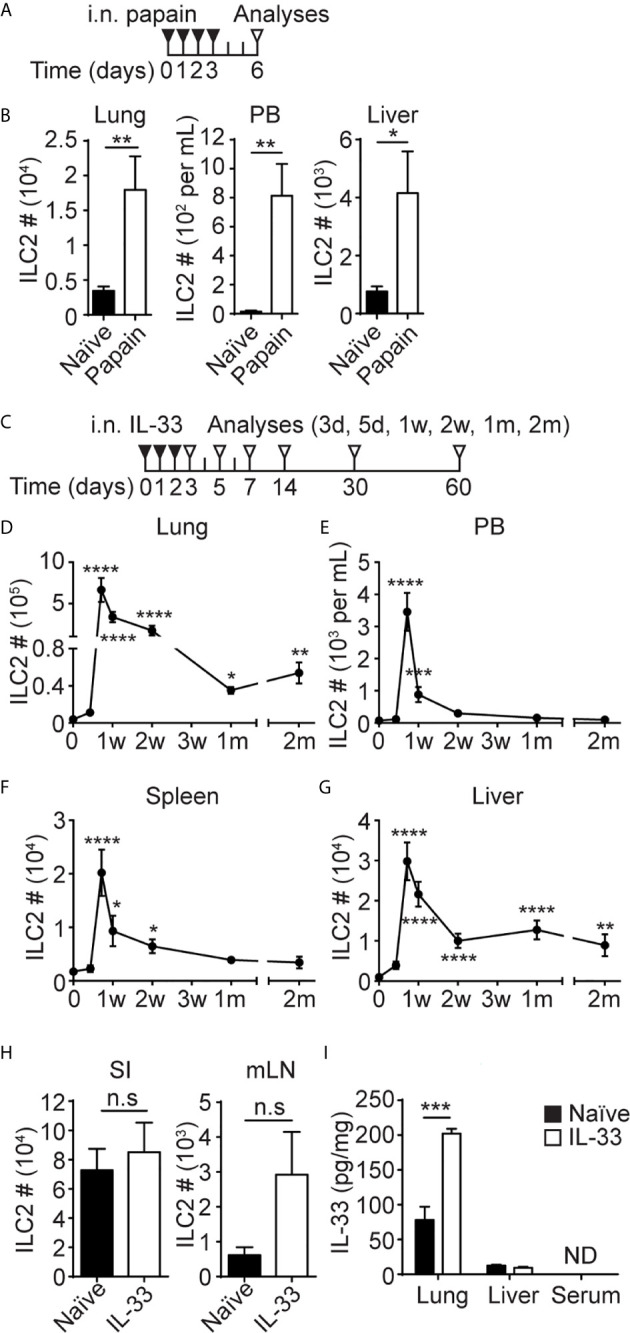
I.n. papain or IL-33 administration causes an increase in ILC2 numbers in the lung, PB and liver. **(A)** Mice received four daily i.n papain injections and were analyzed on day 6. **(B)** ILC2s were quantified in the lung, PB and liver. Black bar=naïve, white bar = papain treated. **(C)** Mice received three daily i.n injections of IL-33 and were analyzed at various time points. d = day, w = week, m = month. **(D–G)** The number of ILC2s in the lung **(D)**, PB **(E)**, spleen **(F)** and liver **(G)** after i.n IL-33 administrations. Day 0 is naïve. Asterisks indicate significant differences from naïve. **(H)** Mice received three daily i.n injections of IL-33 and ILC2s were quantified in the small intestine (SI) and mLN on day 7 [see **(C)**]. **(I)** Mice received three daily i.n. injections of IL-33 and the amount of IL-33 was measured in lung and liver homogenate and serum on day 3 [see **(C)**]. Black bar = naïve, white bar = IL-33 treated, ND = not detected **(H, I)**. Data shown are mean ± SEM. n = 5-10 **(B)**, n = 4-48 **(D–G)**, n = 6 **(H)**, n = 4 **(I)**. Unpaired two-tailed t test **(B, H, I)** or Kruskal-Wallis test **(D–G)** was used to determine statistical significance. *P ≤ 0.05, **P ≤ 0.01, ***P ≤ 0.001, ****P ≤ 0.0001, n.s, not significant (P < 0.05).

We anesthetized mice by isoflurane inhalation during i.n. injections to minimize animal suffering and distress and monitored them until they were fully recovered from anesthesia in a separate cage with a heating pad underneath it. The health status of the mice was assessed daily during i.n. treatments and one day after the last injections based on their behavior, appearance, hydration status, respiration, and presence/absence of any obvious pain. In addition, mice were weighed and closely monitored daily after i.v. ConA injections. Their health status and well-being were assessed daily by facility staff based on their appearance and behavior. At the time of harvest, we anesthetized mice by isoflurane inhalation until they were unconscious and performed euthanasia by carbon dioxide asphyxiation. Female mice were used for most experiments and the mixture of male and female mice was used for CD127 cKO mice characterization (**Supplementary Figures 6C–G**) and *Rorc* ^-/-^ analyses (**Figure 3D**). Mice were used at 6-24 weeks of age and they were randomly assigned to each experimental group. The majority of experiments except those shown in **Figures 1B** (PB), **1L**, **4B, C** (IL-25/IL-33 + TSLP/IL-7 and IL-2/IL-7 only conditions) was performed at least twice to validate the data and each experimental group had at least 3 biological replicates. The detail information is included in each figure legend.

### Generation of CD127 cKO Mice

B6.*RORa-IRES-Cre* (36) mice were re-derived in BCCRC Animal Resource Centre by *in vitro* fertilization of albino C57BL6 (B6(Cg)-Tyr<c-2J>/J) mouse eggs with B6.*RORa-IRES-Cre* mouse sperms obtained from Dr. O’Leary (The Salk Institute). CD127 cKO mice were generated by crossing re-derived B6.*RORa-IRES-Cre* mice and *Il7ra* ^fl/fl^ mice (37), which were kind gifts from Dr. Ninan Abraham (University of British Columbia) with Dr. Singer’s approval.

### Parabiosis

Parabiosis mice were prepared using female B6 and Pep3b mice as previously described (38). Briefly, weight matched female mice were co-housed for 1 week prior to the surgery. The two mice were juxtaposed and the hindlimbs, bodies and forelimbs were joined together, while being maintained in a surgical plane of anesthesia by isoflurane inhalation. Both mice were given subcutaneous injections of 1 mL saline twice a day and 0.03 mg/mL of buprenorphine analgesic for the first 3 days post-surgery. Mice were monitored twice a day during the first week and once a day after that for a total of 3 weeks post-surgery, and the monitoring was extended if mice did not recover well. The health status was assessed based on pain, hydration and body weight loss. Mice were used between 5-6 weeks after the surgery.

### Primary Leukocyte Preparation

Single cell suspensions were prepared from the lung, liver, small intestine, BM and mesenteric LN as previously described (39).

For Kupffer cells and macrophage analyses, livers were mashed through 40 μm cell strainers with 10 mL DMEM + 10% FBS + 100 U/mL P/S + 50 μM 2-ME and the strainers were washed twice with 15 mL of the same media. Hepatocytes were sedimented by centrifugation (4°C, 3 min, 30 × g) and discarded. The supernatant was collected and centrifuged (4°C, 5 min, 300 × g) and the pellet was washed once with 10 mL DMEM + 10% FBS + 100 U/mL P/S + 50 μM 2-ME (4°C, 5 min, 300 × g) before proceeding to red blood cell (RBC) lysis with 150 mM ammonium chloride solution.

To prepare single cell suspension from PB, cardiac blood was collected in PBS containing 25 mM EDTA and cells were pelleted by centrifugation (4°C, 5 min, 600 × g). RBCs were lysed with 150 mM ammonium chloride solution twice.

For spleen, samples were mashed through 70 µm strainers in 5 mL PBS + 2% FBS. Strainers were washed with 5 mL of the same buffer and cells were pelleted by centrifugation (4°C, 5 min, 400 × g). RBCs were lysed using 150 mM ammonium chloride solution.

### Antibodies, Reagents and Flow Cytometers

Isolated leukocytes were counted using a hemocytometer, incubated in 2.4G2 mAb to block Fc receptors for analyses by fluorescence-activated cell sorting (FACS). Fluorescein isothiocyanate (FITC)-conjugated anti-mouse NK1.1 (PK136), TCRβ (H57-597), TCRγδ (GL3), PerCP-Cy5.5-conjugated anti-mouse Ly6C (AL-21), Allophycocyanin (APC)-conjugated anti-mouse CD3ϵ (145-2C11), rat IgG1, κ isotype control (R3-34), Alexa Fluor (AF) 647-conjugated anti-mouse CCR3 (83103), V500-conjugated anti-mouse CD45 (30-F11), CD45.2 (104), Brilliant Violet (BV) 605-conjugated anti-mouse CD90.2 (53-2.1), BV711-conjugated anti-mouse/rat CD49a (Ha31/8), Phycoerythrin (PE)-conjugated anti-mouse CD11b (M1/70), CD19 (1D3), SiglecF (E50-2440), and rat IgG2b, κ isotype control (A95-1) were purchased from BD Biosciences. FITC-conjugated anti-mouse CCR2 (SA203G11), NKp46 (29A1.4), rat IgG2b, κ isotype control (RTK4530), PerCP-Cy5.5-conjugated anti-mouse ITGαL (M17/4), anti-mouse/rat ITGβ1 (HMβ1-1), APC-conjugated anti-mouse CD4 (GK1.5), CD64 (X54-5/7.1), CXCR3 (CXCR3-173), ITGα4 (R1-2), TCRβ (H57-597), TCRγδ (GL3), Armenian hamster IgG isotype control (HTK888), anti-mouse/rat ITGβ3 (2C9.G2, HMβ3-1), AF647-conjugated anti-mouse CD49b (DX5), AF700-conjugated anti-mouse CD45.1 (A20), BV421-conjugated anti-mouse CD5 (53-7.3), BV605-conjugated anti-mouse Ly6G (1A8), BV650-conjugated anti-mouse CD4 (GK1.5), F4/80 (BM8), BV711-conjugated anti-mouse CD103 (2E7), CD19 (6D5), Armenian hamster IgG isotype control (HTK888), PE-conjugated anti-mouse CCR1 (S15040E), CX3CR1 (SA011F11), ITGαV (RMV-7), ITGβ2 (M18/2), anti-mouse/human ITGβ7 (FIB27), rat IgG1, κ isotype control (RTK2071), PECy7-conjugated anti-mouse CCR4 (2G12), and CD206 (C068C2) were purchased from BioLegend. FITC-conjugated anti-mouse T1/ST2 (DJ8) was purchased from MD Biosciences. AF700-conjugated anti-mouse IL-17RB (752101) was purchased from R & D systems. FITC-conjugated anti-mouse CD11b (M1/70), CD19 (1D3), CD3ϵ (145-2C11), FcϵR1a (MAR-1), anti-mouse/rat Ki-67 (SolA15), rat IgG2a, κ isotype control (eBR2a), PerCP-Cy5.5-conjugated CD19 (1D3), CD25 (PC61.5), NK1.1 (PK136), PerCP-eFluor 710-conjugated anti-mouse CD117 (2B8), CD218a (P3TUNYA), CXCR6 (DANIB2), EOMES (Dan11mag), T1/ST2 (RMST2-2), rat IgG2a, κ isotype control (eBR2a), APC-conjugated anti-mouse CCR7 (4B12), CCR9 (CW-1.2), CD103 (2E7), CD8a (53-6.7), KLRG1 (2F1), mouse IgG2a, κ isotype control (eBM2a), eFluor 660-conjugated anti-mouse/human GATA3 (TWAJ), AF700-conjugated anti-mouse CD11c (N418), CD127 (A7R34), CD45.2 (104), eFluor 450-conjugated CD11b (M1/70), CD11c (N418), CD19 (1D3), CD3ϵ (145-2C11), CD4 (RM4-5), Gr-1 (RB6-8C5), MHCII (M5/114.15.2), NK1.1 (PK136), NKp46 (29A1.4), TCRβ (H57-597), TCRγδ (GL3), Ter119 (TER-119), anti-mouse/human CD45R (RA3-6B2), PE-conjugated anti-mouse CD127 (A7R34), CD80 (16-10A1), CXCR4 (2B11), IL-13 (eBio13A), IL-17RB (MUNC33), IL-6 (MP5-20F3), RORγt (B2D), anti-mouse/human GATA3 (TWAJ), PECy7-conjugated anti-mouse CD127 (A7R34), CD24 (M1/69), CD4 (GK1.5), IL-13 (eBio13A), NK1.1 (PK136), and anti-mouse/human T-bet (4B10) were purchased from Thermo Fisher Scientific.

BD FACS Aria was used for cell sorting and BD LSRFortessa was used for flow cytometric analyses. Samples were first gated on live cells using eFluor 780 fixable viability dye (Thermo Fisher Scientific) and then gated on single cells using FSC-H and FSC-A. ILC2s were identified as Lin^-^Thy1^+^ST2^+^CD127^+^ cells (**Supplementary Figure 1A**) in most tissues except small intestine and mesenteric LN, where ILC2s were identified as Lin^-^Thy1^+^CD127^+^GATA3^+^ cells (**Supplementary Figure 1B**). Lymphocyte populations shown in **Figure 5** and **Supplementary Figure 6**, and myeloid populations shown in **Figures 5**–**7** and **Supplementary Figure 6** were identified as shown in **Supplementary Figures 1C** and **2**, respectively. Flowjo version 10.0.7r2 was used for data analyses.

### Intracellular Staining

Leukocytes were incubated at 37°C for 3 hours in 500 µL RPMI 1640 media containing 10% FBS, 100 U/mL P/S, 50 μM 2-ME, Brefeldin A (Golgi Plug, BD Biosciences), 30 ng/mL phorbol 12-myristate 13-acetate (PMA, Sigma, P1585) and 500 ng/mL ionomycin (Sigma, 10634). Intracellular cytokine staining was performed after the incubation and surface staining using Cytofix/Cytoperm Fixation/Permeabilization Solution kit (BD Biosciences) according to manufacturer’s protocol. Intracellular CD206 and CD80 staining was carried out similarly without pre-incubation. Transcription factor and Ki67 staining was performed without pre-incubation using Foxp3/Transcription Factor Staining Buffer Set (Thermo Fisher Scientific) according to manufacturer’s protocol.

### ILC Enrichment

Primary leukocyte cell suspension was enriched for ILCs using EasySep Mouse Pan-ILC Enrichment Kit (STEMCELL Technologies) according to manufacturer’s protocol.

### *In Vivo* Stimulation

Mice were anesthetized by isoflurane inhalation and i.n. injections were given. Mice received i.n. administrations of 0.25 µg IL-33 (BioLegend) or 0.218U papain (Sigma) in 40 µL PBS. For Concanavalin A (ConA, Sigma) treatment, mice were i.v. injected with 7 mg/kg of ConA at 5 mL/kg.

### *In Vivo* Antibody Labeling

Mice were given 3 daily i.n. IL-33 injections as described above. 72 hours after the last treatment, mice were i.v. injected with 2 µg V500-conjugated anti-mouse CD45 antibody. Mice were euthanized 5 minutes after the i.v. antibody injection and the livers were harvested.

### ILC2 Transplantation

Single cell suspensions were prepared from IL-33 treated B6 (WT), *Il6* ^-/-^ or *Cxcr6* ^gfp/gfp^ mouse lungs, followed by ILC enrichment using pan-ILC enrichment kit as described above. ILC2s were quantified by flow cytometry after ILC enrichment and 150,000 (**Supplementary Figures 5A, B**) or 300,000 (**Supplementary Figures 7C–E**) ILC2s were i.v. injected into Pep3b mice.

### FTY720 Treatment

Mice were treated daily with i.n. IL-33 injections as described above. They also received i.p. injections of FTY720 (1 mg/kg) during the IL-33 treatment and for two additional days. One day after the last FTY720 treatment, mice were euthanized to collect tissues for flow cytometry analyses.

### *In Vitro* Stimulation

ILC2s were sorted from lung and liver after ILC enrichment and 1000 cells were cultured (37°C, 5% CO_2_) in 200 µL RPMI1640 media + 10% FBS + 100 U/mL P/S + 50 μM 2-ME, containing 10 ng/mL of IL-33 (BioLegend) and IL-2 (Peprotech), IL-7 (BioLegend), IL-25 (BioLegend) or TSLP (Thermo Fisher Scientific), or 30 ng/mL PMA (Sigma) and 500 ng/mL ionomycin (Sigma). Culture supernatant was collected 72 hours later.

### Quantification of Cytokines

Lung and liver culture supernatant was analyzed for GM-CSF, IL-2, IL-4, IL-6, IL-9, IL-10, TNFα by U-plex assay platform (Meso Scale Discovery), IL-5, IL-13 (Thermo Fisher Scientific) and amphiregulin (R&D systems) by ELISAs.

### Tissue Homogenate and Serum Preparation and Analyses

Lungs and livers were collected from naïve and IL-33 treated mice and homogenized in HBSS with EDTA and Halt protease inhibitor cocktail (Thermo Fisher Scientific, 87786) at 200 mg/mL, following manufacturer’s protocol. The supernatant was analyzed for IL-33 by ELISA (Thermo Fisher Scientific) and total protein contents by Protein Quantification Kit-Rapid (Sigma) according to manufacturer’s protocols. For IL-33 measurement in the serum, blood was collected by cardiac puncture and clotted at room temperature for 30 minutes to an hour. The samples were centrifuged at 2,000 × g for 15 minutes at 4°C and serum (the clear layer at the top) was collected for IL-33 measurement.

### RNA Extraction

15 – 35 mg of median lobe of the liver was homogenized using a pestle. Total RNA was extracted using TRIzol reagent (Thermo Fisher Scientific, 15596018) according to manufacturer’s protocol.

### cDNA Synthesis

cDNA was prepared from 1.5 µg RNA using high-capacity cDNA reverse transcription kit (Thermo Fisher Scientific) according to manufacturer’s protocol.

### qPCR

qPCR was carried out by StepOnePlus system (Thermo Fisher Scientific) using TaqMan fast universal PCR master mix (Thermo Fisher Scientific). Seventy-five ng of cDNA was used per reaction. Changes in gene expression were analyzed relative to naïve samples using 2^-ΔΔC^_T_ method (40). *Tbp* was used as a housekeeping gene for normalization. The following probes/primers were used for detection: *Col1a1* (IDT Assay ID – Mm.PT.58.7562513), Probe – 5’-/56-FAM/CCGGAGGTC/ZEN/CACAAAGCTGAACA/3IABkFQ/-3’, Primer 1 – 5’-CGCAAAGAGTCTACATGTCTAGG-3’, Primer 2 – 5’-CATTGTGTATGCAGCTGACTTC-3’; *Acta2* (IDT Assay ID – Mm.PT.58.16320644), Probe – 5’-/56-FAM/CCGCTGACT/ZEN/CCATCCCAATGAAAGA/3IABkFQ/-3’, Primer 1 – 5’-GAGCTACGAACTGCCTGAC-3’, Primer 2 – 5’-CTGTTATAGGTGGTTTCGTGGA-3’; *Timp1* (IDT Assay ID – Mm.PT.58.30682575), Probe – 5’-/56-FAM/AATCAACGA/ZEN/GACCACCTTATACCAGCG/3IABkFQ/-3’, Primer 1 – 5’-AGACAGCCTTCTGCAACTC-3’, Primer 2 – 5’-CAGCCTTGAATCCTTTTAGCATC-3’; *Tbp* (IDT Assay ID – Mm.PT.39a.22214839), Probe – 5’-/56-FAM/ACTTGACCT/ZEN/AAAGACCATTGCACTTCGT/3IABkFQ/-3’, Primer 1 – 5’-TGTATCTACCGTGAATCTTGGC-3’, Primer 2 – 5’-CCAGAACTGAAAATCAACGCAG-3’.

### Histology

Median lobe of the liver was fixed in 10% formalin for >72 hours and later stored in 70% ethanol. Formalin fixed paraffin embedded sections were prepared and stained with Hematoxylin and Eosin (H&E) staining by Wax-it Histology Services Inc (Vancouver, Canada). Sections were also stained for collagen fibers by picrosirius red stain kit (Abcam, ab150681) according to manufacturer’s protocol.

### Quantification of Picrosirius Red Positive Areas and Granulocyte Clusters

The percentage of picrosirius red staining positive area was determined using ImageJ 1.53e (41). Five areas of each section were analyzed. The H&E stained liver sections of each biological replicate were used to quantify eosinophil clusters.

### Quantification and Statistical Analysis

GraphPad Prism 7 was used for statistical analyses. Unpaired two-tailed t test was used for the comparison between two experimental groups. For multiple comparisons, one-way ANOVA, Kruskal-Wallis test, or two-way ANOVA was used to determine statistical significance, as indicated in each figure. Bonferroni correction was used to adjust the P values. A P-value less than 0.05 was considered significant. *P ≤ 0.05, **P ≤ 0.01, ***P ≤ 0.001, ****P ≤ 0.0001, n.s, not significant (P<0.05). Data shown in figures are mean ± SEM.

## Results

### I.n. Papain or IL-33 Administration Causes an Increase in ILC2 Numbers in the Lung, PB and Liver

To gain insights into ILC2 migration during inflammation, we analyzed their behavior in response to local inflammatory insults in the lung. Consistent with our previous studies (4), i.n. administrations of the protease allergen papain (**Figure 1A**) activated lung ILC2s, and they expanded almost 6-fold (**Figure 1B**, left). The papain treatment also increased the number of ILC2s in PB from less than 100 cells/mL in naïve mice to almost 800 cells/mL (**Figure 1B**, middle), while liver ILC2s also increased by about 6-fold after i.n. papain administration (**Figure 1B**, right). Activation of lung ILC2s by papain is mediated by IL-33 released by epithelial cells (42), so we tested the effects of i.n. administration of recombinant IL-33. We gave three daily i.n. injections of IL-33 into B6 mice and analyzed different tissues at various time points (**Figure 1C**). In agreement with our previous report (20), the number of ILC2s in the lung increased almost 175-fold upon i.n. IL-33 administrations, then contracted but remained high in number for 2 months post injections (**Figure 1D**). The number of ILC2s in PB and spleen similarly increased in the IL-33 treated mice but declined to the naïve mouse levels in 2-4 weeks (**Figures 1E, F**). In the liver, ILC2 numbers peaked on day 5, then slowly decreased but remained higher than those in naïve liver for 2 months (**Figure 1G**). We also quantified ILC2s in the small intestine (SI) and mesenteric lymph nodes (mLN) on day 7. The number of intestinal and mLN ILC2s slightly increased after i.n. IL-33 injections, but the differences were not statistically significant (**Figure 1H**). Quantification of ILC2s in the lung, liver and PB of mice at multiple time points after i.n. papain injections (**Supplementary Figure 3A**) demonstrated similar kinetics of ILC2 expansion as seen after i.n. IL-33 administrations (**Supplementary Figure 3B**). However, ILC2s contracted more quickly and their numbers in the liver returned to the naïve levels two weeks after the initial activation by papain treatment.

Since the liver is a highly vascularized organ, we performed *in vivo* antibody labeling to determine whether ILC2s are in the vasculature or in the liver tissue. Even at the peak of ILC2 circulation, only 2.5% of the ILC2s isolated from the liver were labeled, unlike circulating cells such as NK and T cells (**Supplementary Figure 1C**). Therefore, the majority of ILC2s are in tissues rather than in circulation.

To determine whether i.n. administered IL-33 entered circulation and caused systemic activation of ILC2s, we measured IL-33 in tissue homogenate prepared from the lung and liver as well as serum of the IL-33 treated mice one day after three daily i.n. injections. The amount of IL-33 increased in the lung after the IL-33 treatment as expected but did not increase in the liver and was undetectable in serum (**Figure 1I**). These results suggest that the expansion of the ILC2 populations in PB, spleen and the liver in the i.n. IL-33 administered mice was unlikely due to systemic activation of ILC2s. Notably, we did detect an increase in the frequency of Ki67^+^ ILC2s in the BM of IL-33 treated mice compared to naïve mice (**Supplementary Figure 3D**), suggesting possible systemic effects of IL-33. However, it was also possible that activated Ki67^+^ ILC2s migrated into the BM from the lung.

### ILC2s Migrate From the Lung to the Liver Upon i.n. IL-33 Treatment

**Figure 2:**
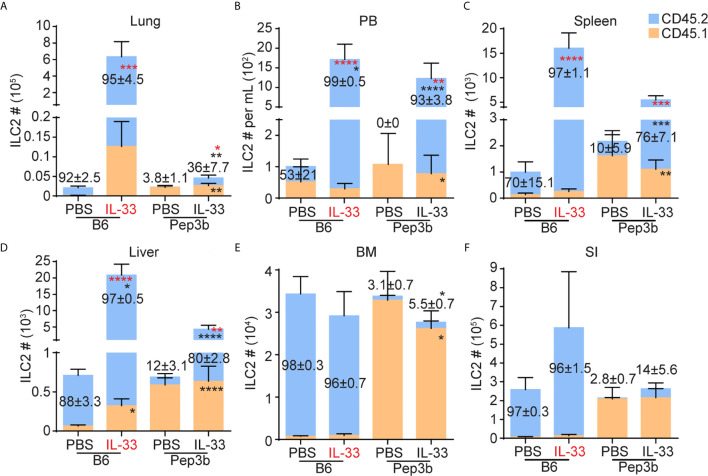
ILC2s migrate from the lung to the liver upon i.n. IL-33 treatment. B6 and Pep3b mice were conjoined by parabiosis surgery. B6 mice were given three daily i.n. PBS or IL-33 injections and both B6 and Pep3b mice were analyzed on day 7 (see **Figure 1C**). CD45.1^+^ and CD45.2^+^ ILC2s were quantified in the lung **(A)**, PB **(B)**, spleen **(C)**, liver **(D)**, BM **(E)** and SI **(F)**. X-axes show the strains of mice analyzed (B6 or Pep3b) and treatment groups (PBS or IL-33). IL-33 highlighted in red indicates the mouse that received IL-33 injections. Numbers within graphs indicate the percentages of CD45.2^+^ ILC2s. Black and red asterisks indicate statistical significance of percentages and cell numbers, respectively, of CD45.2^+^ (within blue bars) and CD45.1^+^ (within orange bars) ILC2s compared to PBS treated pairs. Data shown are mean ± SEM. n = 6-7 (except SI, where n = 3-4). Two-way ANOVA with Bonferroni correction was used to determine statistical significance. *P ≤ 0.05, **P ≤ 0.01, ***P ≤ 0.001, ****P ≤ 0.0001.

To determine whether i.n. administration of IL-33 induces proliferation of lung-resident ILC2s and their migration to the liver through PB, we generated parabiotic mice by conjoining CD45.2^+^ B6 and CD45.1^+^ Pep3b mice. Five to six weeks after the surgery, we gave B6 mice three daily i.n. injections of IL-33 (or PBS as a control) and analyzed various tissues from both the B6 and Pep3b mice on day 7 (**Supplementary Figure 4A**). In PBS treated control mice, most (more than 92%) ILC2s in the lung, BM and SI were of host origin in both B6 and Pep3b mice as previously reported (28, 29), whereas smaller fractions of ILC2s in PB and spleen were host-derived, although the percentages varied due to small numbers of ILC2s in PBS control mice (**Figures 2A–F**). Upon i.n. IL-33 administration into B6 mice, both CD45.1^+^ and CD45.2^+^ ILC2s expanded in the lung of B6 mice while their ratio remained unchanged from control mice, indicating an expansion of lung resident ILC2s rather than recruitment from other tissues. In the Pep3b partners, the number of lung ILC2s slightly increased due to an increase in CD45.2^+^ cells while CD45.1^+^ ILC2 numbers did not significantly change (**Figure 2A**). ILC2 numbers in PB and spleen also increased in both B6 and Pep3b mice, and the increases were almost entirely accounted for by the increases in CD45.2^+^ ILC2s (**Figures 2B, C**). In the liver of B6 mice, ILC2 numbers significantly increased in response to IL-33 treatment due to a large increase in CD45.2^+^ ILC2s while the percentage of CD45.1^+^ cells decreased. In Pep3b liver, CD45.2^+^, but not CD45.1^+^, ILC2 numbers significantly increased (**Figure 2D**). IL-33 administration had little effect on ILC2s in the BM and SI of both B6 and Pep3b mice (**Figures 2E, F**). The i.n. IL-33 treatment had no effect on the total numbers of B cells in the lung or liver and the ratio between CD45.1^+^ and CD45.2^+^ remained 1:1 as expected for circulating cells (**Supplementary Figures 4B, C**). To exclude the possibility that genetic differences between B6 and Pep3b mice might contribute to those results, we also administered i.n. IL-33 into Pep3b instead of B6 mice. The results were very similar to those with IL33 treatment into B6 mice (**Supplementary Figures 4D–K**). These results together demonstrate that lung resident ILC2s locally expand upon i.n. IL-33 treatment rather than being recruited from elsewhere, and some activated lung ILC2s leave the lung, circulate in blood and mostly settle in the liver.

### Liver ILC2s Phenotypically Differ From Lung ILC2s

**Figure 3:**
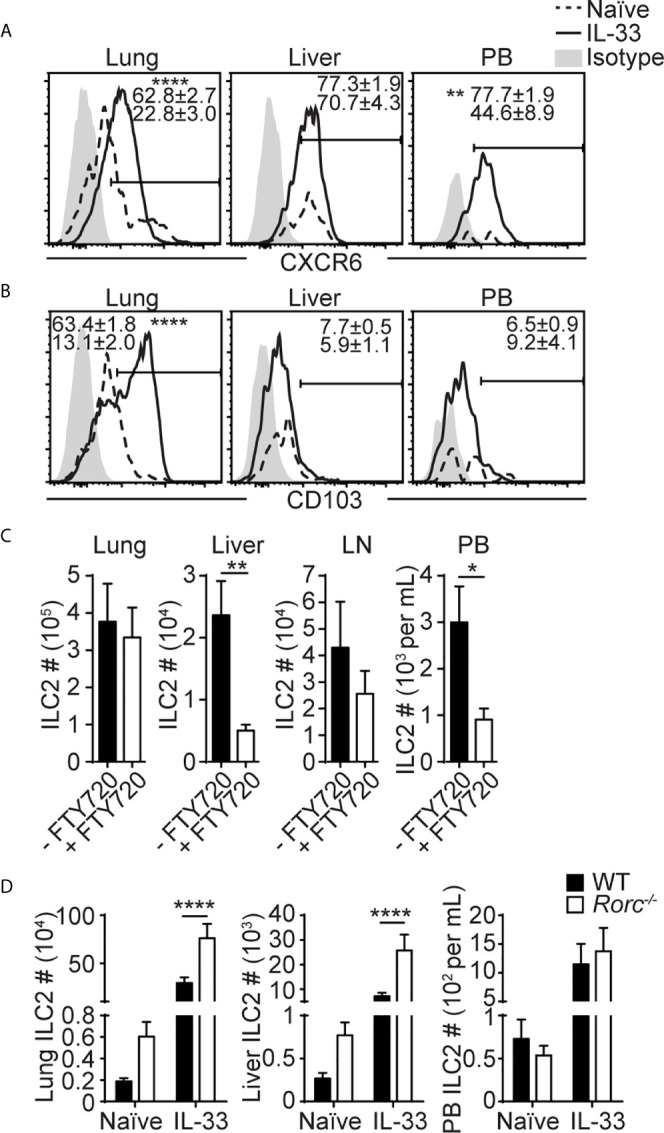
Liver ILC2s phenotypically differ from lung ILC2s. **(A, B)** Mice received three daily i.n IL-33 injections and CXCR6 **(A)** and CD103 **(B)** expression was analyzed before and 3 days after IL-33 treatment (day 5 in **Figure 1C**). Histograms show representative expression of each marker. Numbers show percentages of CXCR6^+^ **(A)** or CD103^+^ **(B)** ILC2s. Histograms: dotted lines/numbers at the bottom = naïve, solid lines/numbers on top = IL-33 treated, shaded = isotype. **(C)** Mice were administered with i.n. IL-33 on day 0-2 and i.p. FTY720 on day 0-4, and ILC2s were quantified in the lung, liver, mediastinal LN, and PB on day 5. **(D)** ILC2s were quantified in WT (black bars) and *Rorc* ^-/-^ (white bars) lung, liver and PB before and 5 days after three daily i.n. IL-33 injections (day 7 in **Figure 1C**). Data shown are mean ± SEM. n = 6-15 **(A)**, n = 5-23 **(B)**, n = 6 **(C)**, n = 5-19 **(D)**. Unpaired two-tailed t test **(A–C)** or two-way ANOVA with Bonferroni correction **(D)** was used to determine statistical significance. *P ≤ 0.05, **P ≤ 0.01, ****P ≤ 0.0001.

To characterize lung-derived migratory ILC2s, we compared ILC2s in the lung, PB and liver before and after i.n. IL-33 administration. They differed in the expression of activation-associated ILC2 markers, including KLRG1, IL-25R, integrins and chemokine receptors as summarized in **Table 1**. Among them, the chemokine receptor CXCR6 and the integrin alpha-chain CD103 (αE) were of particular interest. In naïve mice, CXCR6, which is required for some lymphocytes to enter the liver (43), was highly expressed on ~25% of lung ILC2s, ~50% of PB ILC2s and ~70% of liver ILC2s. After i.n. IL-33 administration, ILC2s in all the tissues tested became uniformly positive for CXCR6 (**Figure 3A**). CD103 paired with the β7 chain is known to bind E-cadherin and mediate the localization of intraepithelial lymphocytes (44). Almost all lung ILC2s in naïve mice were CD103^lo^. About 65% of IL-33 activated lung ILC2s highly expressed CD103 while ~35% remained CD103^lo^. In contrast, PB and liver ILC2s were mostly CD103^lo^ regardless of the i.n. IL-33 treatment (**Figure 3B**).

**Table 1:** Summary of surface marker expressions on naïve and IL-33 treated (+IL-33) lung and liver ILC2s.

	Lung		Liver	
	Naïve	+IL-33^a^	Naïve	+IL-33
CD25	+^b^	15% +	70% +	55% +
KLRG1	40% +	70% +	40% +	+
IL25R	40% +	75% +	60% +	+
CX3CR1	–^c^	–	–	–
CCR7	–	–	–	–
CCR3	–	–	–	–
CCR4	10% +	–	10% +	–
CXCR4	20% +	15% +	–	–
CCR2	–	–	–	–
CXCR3	–	–	–	–
CCR1	–	–	–	–
CXCR6	25% +	60% +	70% +	75% +
CCR9	–	–	30% +	–
ITGαE	15% +	65% +	–	–
ITGβ7	+	+	75% +	+
ITGαL	+	+	+	+
ITGβ1	65% +	70% +	75% +	80% +
ITGβ3	+	+	+	+
ITGαv	+	+	+	+
ITGβ2	+	+	+	+
ITGα4	45% +	80% +	65% +	+

^a.^ +IL-33 mice were given three daily i.n. injections of IL-33 and analyzed between day 5 - 7.

^b.^ “+” indicates >80% of the cells express the markers.

^c.^ “-” indicates <10% of the cells express the markers.

To test the role of CXCR6 in the migration of ILC2s to the liver, we transplanted IL-33 treated B6 wild-type (WT) or CXCR6-deficient (*Cxcr6* ^gfp/gfp^) lung ILC2s (CD45.2^+^) into Pep3b mice (CD45.1^+^) and quantified the donor ILC2s in the lung and liver of the Pep3b mice (**Supplementary Figure 5A**). We chose to transplant activated ILC2s instead of analyzing IL-33 treated WT and *Cxcr6* ^gfp/gfp^ mice because naïve and papain treated *Rag* ^-/-^ *Cxcr6* ^gfp/gfp^ mice have been shown to have reduced numbers of ILC2s compared to *Rag* ^-/-^ mice (45). While mice transplanted with *Cxcr6* ^gfp/gfp^ ILC2s trended towards reduced numbers of donor ILC2s in the lung and liver compared to those given WT ILC2s, the difference was not significant (**Supplementary Figure 5B**), suggesting that CXCR6 may be involved, but it is not necessary for ILC2 migration to the liver.

CD103 (integrin αE) pairs with the integrin β7 and interacts with E-cadherin on epithelial cells (44). To determine the role of CD103 in the retention of ILC2s in the lung, we quantified ILC2s in the lung, liver and PB of WT and *Itgb7* ^-/-^ mice. Without IL-33 treatment, there was no significant difference in ILC2 numbers between WT and the mutant mice (**Supplementary Figure 5C**). Upon IL-33 treatment, significantly more ILC2s were detected in the lungs and PB of *Itgb7* ^-/-^ compared to WT mice, while the numbers were similar in the liver. The ratio of PB to lung ILC2 numbers was similar in WT and *Itgb7* ^-/-^ mice (**Supplementary Figure 5D**). These results suggest that CD103 may negatively regulate lung ILC2 activation and/or proliferation but unlikely regulate ILC2 emigration from the lung.

Previous studies have demonstrated that the migration of intestinal ILC2s stimulated by IL-25 or parasite infection is S1P dependent (33, 34). Therefore, we tested whether IL-33 induced ILC2 migration from the lung to the liver is also dependent on S1P. Administration of FTY720, which binds to S1P receptors to induce their internalization and degradation, during and after IL-33 treatment resulted in marked reduction in ILC2 numbers in the liver and PB, while they remained unchanged in the lung and the draining mediastinal LN (**Figure 3C**). These results demonstrate that ILC2s migrate from the lung to the liver in a S1P receptor-dependent manner upon IL-33 treatment.

We have previously found that i.n. administration of IL-33 resulted in a large increase in ILC2 numbers in the lung draining mediastinal LN (20), suggesting that activated lung ILC2s migrate into mediastinal LN before they enter PB. Therefore, we analyzed the migration of lung ILC2s in *Rorc^-/-^* mice that lack LNs (46) to determine whether the LNs were required for ILC2 migration. The numbers of ILC2s in the naïve lung and liver were higher in *Rorc* ^-/-^ than WT mice (**Figure 3D**). Upon i.n. IL-33 treatment, ILC2 numbers in the lung, liver and PB increased in both *Rorc* ^-/-^ and WT mice, and ILC2 numbers in the lung and liver, but not PB, were slightly higher in *Rorc* ^-/-^ mice than WT mice, indicating that LNs are not required for the migration of ILC2s from the lung to the liver. These results suggest that a subset of activated lung ILC2s exits the lung and enters the blood independent of LNs and preferentially settles in the liver.

### Lung-Derived Liver ILC2s Produce More IL-5, IL-13 and IL-6 Than Lung ILC2s

**Figure 4:**
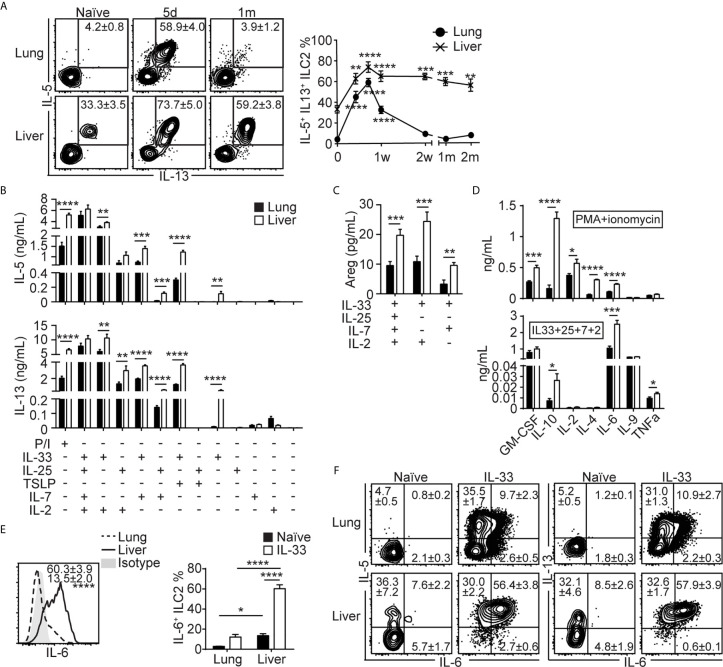
Lung-derived liver ILC2s produce more IL-5, IL-13 and IL-6 than lung ILC2s. **(A)** Mice received three daily i.n IL-33 injections and lung and liver were analyzed for intracellular cytokine expressions at various time points as shown in **Figure 1C**. The plots show IL-5 and IL-13 expressions in the lung (top) and liver (bottom) ILC2s at three selected time points. Boxes indicate IL-5^+^IL-13^+^ ILC2s and the numbers show mean ± SEM percentages of IL-5^+^IL-13^+^ ILC2s. The graph shows the IL-5^+^IL-13^+^ lung (filled circle) and liver (cross) ILC2 percentages. d = day, w = week, m = month. Asterisks indicate significant differences compared to naïve. **(B–D)** Mice were treated as above and ILC2s were purified on day 14 (see **Figure 1C**) and cultured for 72 hours. The amounts of IL-5 [**(B)**, top], IL-13 [**(B)**, bottom], amphiregulin (Areg) **(C)** and various cytokines **(D)** in conditions indicated below graphs **(B, C)**, P/I [**(D)**, top] or IL-33 + IL-25 + IL-7 + IL-2 [**(D)**, bottom] conditions. P/I = PMA + ionomycin, “+” = present, “-” = absent. Black = lung, white = liver. **(E, F)** Lung and liver ILC2s were stained for intracellular IL-5, IL-6 and IL-13 before and 5 days after IL-33 treatment (day 7 in **Figure 1C**). **(E)** Histograms (left) show IL-6 expression in IL-33 treated lung (dashed line/number at the bottom) and liver (solid line/number on top) ILC2s. Shaded = isotype. Graphs (right) show IL-6^+^ ILC2 percentages in naïve (black bars) and IL-33 treated (white bars) lung and liver. **(F)** IL-5, IL-6 and IL-13 expressions in the lung (top) and liver (bottom) ILC2s in naïve (left) and IL-33 treated (right) mice. Numbers indicate percentages of ILC2s in cytokine positive quadrants. Data shown are mean ± SEM. n = 3-22 **(A)**, n = 9-15 (except n = 3-5 for IL-25/IL-33 + TSLP/IL-7 and IL-2/IL-7 only conditions) **(B, C)**, n = 7 **(D)**, n = 6 **(E, F)**. One-way **(A)** or two-way **(E)** ANOVA with Bonferroni correction, or unpaired two-tailed t test **(B–D)** was used to determine statistical significance. *P ≤ 0.05, **P ≤ 0.01, ***P ≤ 0.001, ****P ≤ 0.0001.

To investigate functions of lung-derived ILC2s in the liver, we analyzed intracellular cytokine expression in ILC2s in the lung and liver of naïve and IL-33 treated mice at various time points after the treatment. Unlike naïve lung ILC2s, which are mostly negative for IL-5 and IL-13, approximately 33% of naïve liver ILC2s were positive for these cytokines, indicating that some liver ILC2s have the ability to express intracellular IL-5 and IL-13 in steady state (**Figure 4A**). After IL-33 administration, the percentage of cytokine positive lung ILC2s increased significantly until day 5 and quickly decreased, reaching a similar level as in naïve ILC2s by two weeks after the IL-33 treatment. In contrast, the percentages of IL-5 and IL-13 double positive liver ILC2s peaked on day 5, plateaued and remained high until 2 months after the treatment (**Figure 4A**). We purified ILC2s from the lung and the liver 14 days after the IL-33 treatment and stimulated ex vivo with phorbol 12-myristate 13-acetate (PMA) + ionomycin or various combinations of cytokines known to activate ILC2s, and the culture supernatant was analyzed for IL-5 and IL-13. Strikingly, in most conditions tested, ILC2s isolated from the liver produced greater amounts of IL-5 and IL-13 compared to those from the lung, except for the culture stimulated with IL-2 only, where lung ILC2s produced slightly more cytokines than liver ILC2s (**Figure 4B**). Interestingly, ILC2s isolated from the liver produced small amounts of IL-5 and IL-13 upon stimulation with IL-33 only, which did not stimulate lung ILC2s. The amounts of amphiregulin (Areg), a growth factor known to be produced by ILC2s (47), were very low, although ILC2s isolated from the liver produced significantly more than those from the lung (**Figure 4C**). The supernatants collected from PMA + ionomycin and IL-33 + IL-25 + IL-7 + IL-2 conditions were also analyzed by multiplex cytokine array to determine cytokine profiles of the lung and lung-derived liver ILC2s. Upon stimulation with PMA + ionomycin, both lung and liver ILC2s produced detectable amounts of granulocyte-macrophage colony-stimulating factor (GM-CSF), IL-10, IL-2, IL-4 and IL-6 (**Figure 4D**, top). When stimulated with IL-33 + IL-25 + IL-7 + IL-2, both lung and liver ILC2s produced GM-CSF and IL-9, while only small amounts of IL-10, IL-2, IL-4 and TNFα were detected (**Figure 4D**, bottom). Unexpectedly, ILC2s isolated from the liver produced large amounts of IL-6. Intracellular cytokine staining also showed greater percentages of liver ILC2s expressing IL-6 after IL-33 treatment compared to the lung ILC2s (**Figure 4E**). Co-staining of lung and liver ILC2s before and after IL-33 treatment with IL-5, IL-13 and IL-6 showed that the majority of lung and liver ILC2s in naïve mice were negative for IL-6, and most liver ILC2s that became IL-6^+^ by the IL-33 treatment co-expressed IL-5 or IL-13 (**Figure 4F**). In summary, lung-derived liver ILC2s produce more type 2 cytokines, Areg and IL-6 than lung ILC2s.

### Lung-Derived Liver ILC2s Induce Type 2 Skewing in the Liver

**Figure 5:**
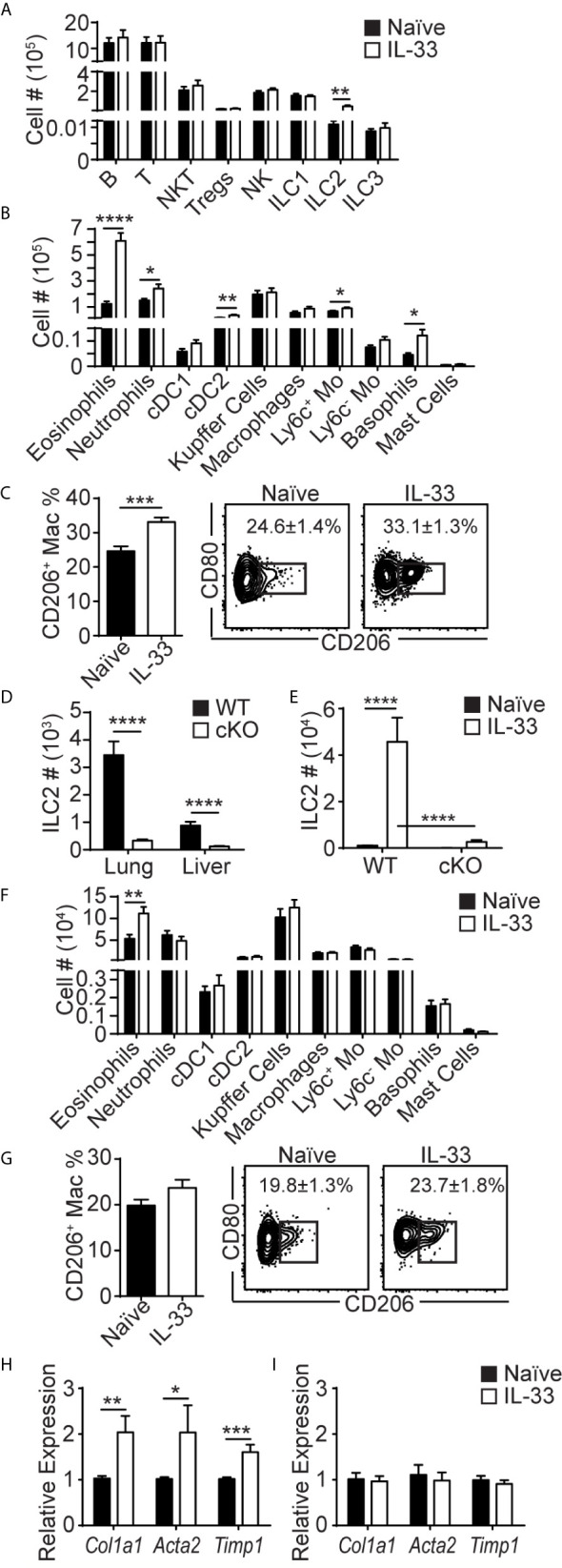
Lung-derived liver ILC2s induce type 2 skewing in the liver. **(A–C)** WT mice received three daily injections of i.n. IL-33 and the livers were analyzed for various lymphocytes **(A)**, myeloid populations **(B)**, and CD206^+^ macrophages **(C)** 5 days later (day 7 in **Figure 1C**). **(D)** ILC2 numbers in the lung and liver of naïve WT (black bars) and CD127 cKO (white bars) mice. **(E)** WT and CD127 cKO mice were treated as above and liver ILC2s were quantified. **(F, G)** CD127 cKO mice were treated as above and the livers were analyzed for myeloid cells **(F)** and CD206^+^ macrophages **(G)**. **(H, I)** WT **(H)** and CD127 cKO **(I)** mice were treated as above and the livers were collected 3 days later (day 5 in **Figure 1C**) and the expression of indicated genes was analyzed. Black bars = naïve, white bars = IL-33 treated except **(D)** Numbers in plots show percentages of CD206^+^ macrophages **(C, G)**. Tregs = regulatory T cells, cDC1 = conventional type 1 dendritic cells (CD103^+^), cDC2 = conventional type 2 DCs (CD11b^+^), Mo = monocytes. Data shown are mean ± SEM. n = 6-17 **(A–C)**, n = 17-21 **(D)**, n = 6 **(E)**, n = 6-18 **(F)**, n = 9-10 **(G)**, n = 5-11 **(H)**, n = 5 **(I)**. Unpaired two-tailed t test **(A–D, F–I)** or two-way ANOVA with Bonferroni correction **(E)** was used to determine statistical significance. *P ≤ 0.05, **P ≤ 0.01, ***P ≤ 0.001, ****P ≤ 0.0001.

To investigate the effects of lung ILC2 migration to the liver, we analyzed various other lymphocytes and myeloid cells in the liver before and 5 days after i.n. IL-33 treatment. Flow cytometric analyses showed that ILC2s were the only lymphocyte population that expanded in the liver upon i.n. IL-33 treatment (**Figure 5A**). Eosinophils expanded greatly, suggesting that lung-derived liver ILC2s actively produced IL-5 (**Figure 5B**). The numbers of neutrophils, CD11b^+^ dendritic cells (cDC2), Ly6c^+^ monocytes and basophils also slightly increased. The number of monocyte-derived macrophages (CD11b^hi^ F4/80^lo^; referred to as macrophages from here on) remained unchanged before and after IL-33 stimulation. However, the percentage of macrophages expressing intracellular CD206 (M2 phenotype) significantly increased after the IL-33 treatment, likely due to IL-13 produced by lung-derived liver ILC2s (**Figure 5C**). Papain administration also caused a significant increase in liver eosinophils and small yet significant increases in macrophages, whereas the percentage of CD206^+^ macrophages remained unchanged (**Supplementary Figures 6A, B**). This suggests that the number of lung-derived ILC2s in the liver after papain injections is sufficient to induce eosinophilia but not macrophage differentiation.

To determine whether ILC2s mediate these changes in myeloid cell populations in the liver, we analyzed the ILC2 deficient CD127 cKO mice newly generated by crossing *Rora*-IRES-Cre (36) and *Il7ra* ^fl/fl^ (37) mice. Although the composition of immune cells was altered in CD127 cKO mice compared to WT mice, ILC2s were the only population that was significantly reduced in all tissues analyzed (**Supplementary Figures 6C–G**). The numbers of lung and liver ILC2s in CD127 cKO mice were about 10% of those in WT mice (**Figure 5D**). Upon IL-33 treatment, the residual ILC2s expanded, but the numbers of ILC2s in the liver of CD127 cKO mice remained about 10% of those in WT mice (**Figure 5E**). I.n. IL-33 injections induced mild eosinophilia in the CD127 cKO mouse liver, likely due to the residual ILC2s, while there was no increase in other myeloid populations (**Figure 5F**), indicating that ILC2s are responsible for the type 2 skewing seen in the liver after i.n. IL-33 treatment. Similarly, the percentages of macrophages expressing CD206 were no longer significantly different (**Figure 5G**). These results suggest that lung-derived ILC2s produce IL-5 and IL-13 in the liver, inducing type 2 skewing.

ILC2s have previously been implicated in tissue remodeling and fibrosis in the liver through production of IL-13 (8). Therefore, we analyzed the expression of the fibrosis-related genes *Col1a1*, *Acta2* and *Timp1* in the liver by quantitative polymerase chain reaction (qPCR). The expression of these genes was significantly increased by i.n. IL-33 administration into WT mice (**Figure 5H**). Strikingly, the increase in the expression of the fibrosis-related genes was diminished in ILC2-deficient CD127 cKO mice (**Figure 5I**). These results indicate that lung-derived liver ILC2s induce type 2 inflammation in the liver and may be involved in development of liver fibrosis.

### Repeated i.n. IL-33 Injections Cause Eosinophilic Inflammation and Expression of Fibrosis-Related Genes in the Liver

**Figure 6:**
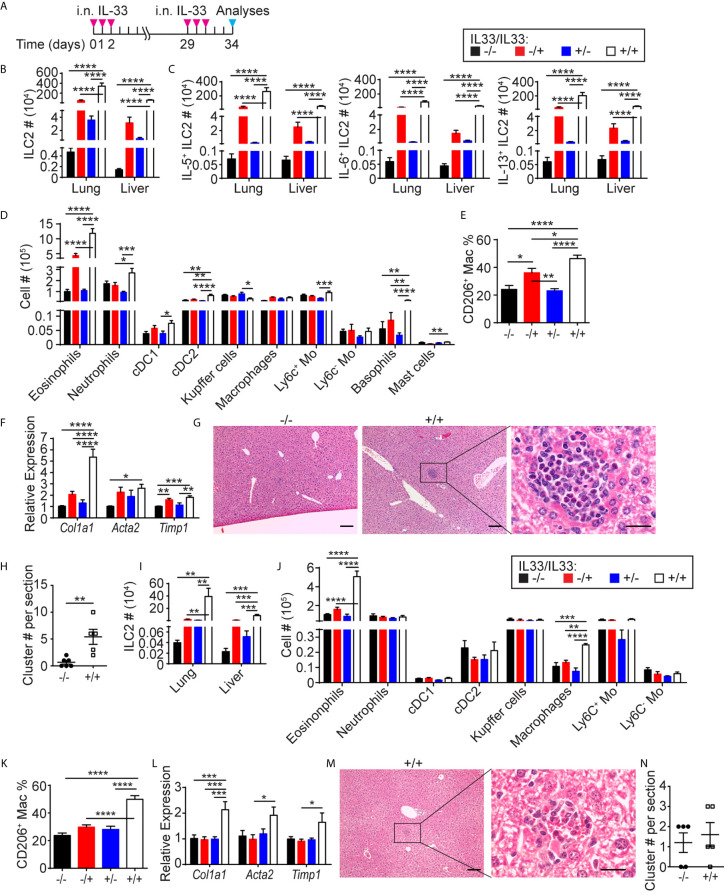
Repeated i.n. IL-33 injections cause eosinophilic inflammation and expression of fibrosis-related genes in the liver. **(A)** WT mice received three daily i.n. IL-33 treatment and challenged with another set of IL-33 injections a month later. Mice were analyzed three days after the last injection. **(B–F)** ILC2s **(B)** and IL-5^+^, IL-6^+^ and IL-13^+^ ILC2s **(C)** were quantified in the lung and liver, and various myeloid populations **(D)** and CD206^+^ macrophages **(E)** were quantified in the liver. The livers were analyzed for expression of indicated genes by qPCR **(F)**. **(G)** H&E staining of -IL33/-IL33 (left, scale bar = 100 µm) and +IL33/+IL33 treated liver at low (middle, scale bar = 100 µm) and high (right, scale bar = 20 µm) magnifications. **(H)** The eosinophilic clusters were quantified in -IL33/-IL33 and +IL33/+IL33 treated liver sections. **(I–N)** CD127 cKO mice were treated as in **(A)**. ILC2s **(I)**, various myeloid populations **(J)**. CD206^+^ macrophages **(K)** and expression of indicated genes **(L)** were quantified in the liver. **(M)** H&E staining of +IL33/+IL33 treated liver at low (left, scale bar = 100 µm) and high (right, scale bar = 20 µm) magnifications. **(N)** The eosinophilic clusters were quantified in -IL33/-IL33 and +IL33/+IL33 treated liver sections. “+/-” indicates presence or absence of the first and second treatments, respectively. Data shown are mean ± SEM. n = 6-12 **(B–F)**, n = 5-6 **(G, H)**, n = 5 **(I–N)**. Two-way ANOVA with Bonferroni correction **(B–F, I–L)** or unpaired two-tailed t test **(H, N)** was used to determine statistical significance. *P ≤ 0.05, **P ≤ 0.01, ***P ≤ 0.001, ****P ≤ 0.0001.

To enhance migration of lung ILC2s to the liver, we gave two sets of i.n. IL-33 administrations one month apart, mimicking chronic type 2 inflammation in the lung (**Figure 6A**). Three days after the last administration, ILC2 numbers in the lung and liver of the mice treated with two sets of IL-33 (+IL33/+IL33) were much higher than those in the mice treated with one set of IL-33 (-IL33/+IL33, +IL33/-IL33) or naïve mice (-IL33/-IL33) (**Figure 6B**). Similarly, IL-5, IL-6 and IL-13 expressing ILC2s also expanded significantly in the lung and liver of +IL33/+IL33 mice compared to -IL33/+IL33, +IL33/-IL33 or -IL33/-IL33 mice (**Figure 6C**). Analyses of myeloid cell populations in the liver revealed that eosinophils, neutrophils, CD103^+^ DC (cDC1), cDC2, Ly6c^+^ monocytes, basophils and mast cells were all increased in +IL33/+IL33 treatment group compared to other groups (**Figure 6D**). Interestingly, the tissue resident Kupffer cells (CD11b^lo^ F4/80^hi^) were slightly decreased in +IL33/+IL33 treatment group in comparison to +IL33/-IL33 group, although it is not clear whether this is due to the change in their phenotype or decrease in their numbers. IL-33 challenge also caused an increase in the frequency of CD206^+^ macrophages (**Figure 6E**). Strikingly, the +IL33/+IL33 treatment greatly enhanced the expression of *Col1a1* gene compared to naïve or single treatment groups, reaching a 5-fold increase in relative expression compared to the naïve liver, whereas *Acta2* or *Timp1* gene expression was comparable to a single treatment group (-IL33/+IL33 or +IL33/-IL33) (**Figure 6F**). Interestingly, H&E staining showed large cell clusters containing eosinophils in the livers of +IL33/+IL33 treated mice (**Figure 6G**). These clusters were much smaller and less frequent in naïve mouse livers (**Figures 6G, H**).

To determine whether fibrosis-associated gene expression and eosinophilic hepatitis were due to ILC2s, we treated CD127 cKO mice with two sets of IL-33 administrations as described above. ILC2s significantly expanded in the lung and liver of the treated mice, while the numbers of ILC2s were substantially lower compared to those in WT mice (**Figure 6I**). Similarly, eosinophils were also elevated after two sets of IL-33 injections, although only to a small degree compared to in WT mice (**Figure 6J**). In contrast, the percentage of CD206^+^ macrophages increased to the same level as in WT mice (**Figure 6K**). The expression of fibrosis-related genes was only mildly elevated (**Figure 6L**). Eosinophilic clusters in the livers of +IL33/+IL33 treated CD127 cKO mice were less frequent and smaller than those in the WT mouse livers (**Figures 6M, N**). These results indicate that lung-derived ILC2s induce eosinophilic inflammation and fibrosis-associated gene expression in the liver.

### I.n. IL-33 Treatment Attenuates ConA-Induced Hepatitis

**Figure 7:**
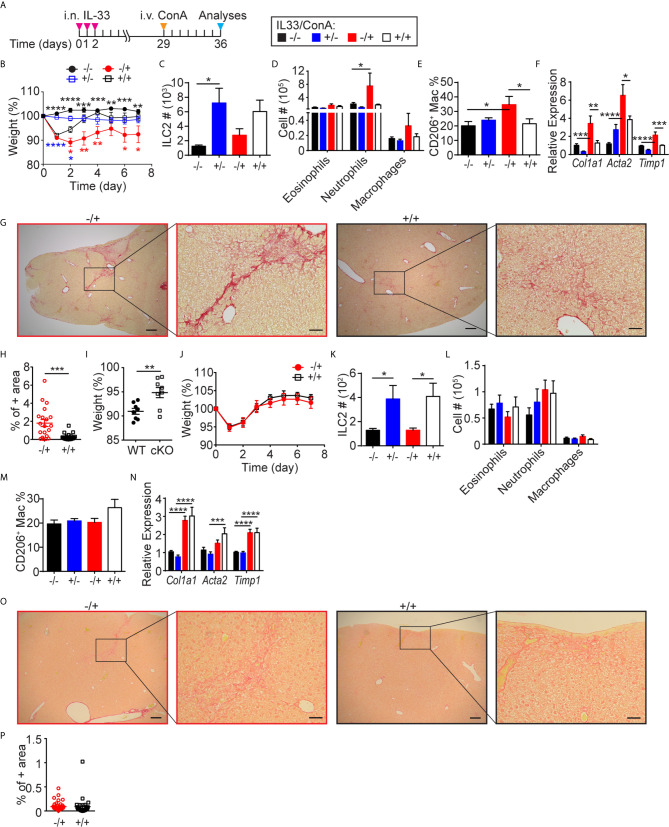
I.n. IL-33 treatment attenuates ConA-induced hepatitis. **(A)** WT mice received three daily i.n. IL-33 treatment and i.v. injected with ConA one month later. Mice were analyzed 1 week after ConA treatment. **(B)** Mice were weighed daily after ConA injections. Black, blue and red asterisks indicate statistically significant differences between -IL33/-ConA *vs.* -IL33/+ConA, +IL33/-ConA *vs.* +IL33/+ConA, and -IL33/+ConA *vs.* +IL33/+ConA, respectively. ILC2s **(C)**, myeloid cells **(D)**, CD206^+^ macrophages **(E)** and transcripts of indicated genes **(F)** were quantified in the liver. **(G)** Picrosirius red staining of -IL33/+ConA (red) and +IL33/+ConA (black) treated livers at low (left, scale bar = 200 µm) and high (right, scale bar = 50 µm) magnifications. **(H)** Picrosirius red+ area was quantified in -IL33/+ConA (red circles) and +IL33/+ConA (black squares) treated livers. **(I–P)** CD127 cKO mice were treated and analyzed as described in **(A)** The percentage body weights of -IL33/+ConA treated WT (circles) and cKO (squares) mice 24 hours after ConA treatment was calculated with respect to the weights at the time of ConA injections **(I)**. Mice were weighed daily after ConA injections **(J)**. ILC2s **(K)**, myeloid cells **(L)**, CD206^+^ macrophages **(M)** and transcripts of indicated genes **(N)** were quantified in the liver. **(O)** Picrosirius red staining of -IL33/+ConA (red) and +IL33/+ConA (black) treated livers at low (left, scale bar = 200 µm) and high (right, scale bar = 50 µm) magnifications. **(P)** Picrosirius red+ area was quantified in -IL33/+ConA (red circles) and +IL33/+ConA (black squares) livers. “+/-” indicates presence or absence of i.n. IL-33 and i.v. ConA treatments, respectively. Data shown are mean ± SEM. n = 4-8 **(B–H)**, n = 6-8 **(I–P)**. Two-way ANOVA with Bonferroni correction **(B–F, K–N)** or unpaired two-tailed t test **(H–J, P)** was used to determine statistical significance. *P ≤ 0.05, **P ≤ 0.01, ***P ≤ 0.001, ****P ≤ 0.0001.

To further investigate the functional role of lung-derived ILC2s in the liver, we tested their effects on ConA-induced acute hepatitis. We gave three consecutive days of i.n. IL-33 injections followed by a single i.v. injection of ConA one month later (**Figure 7A**). Both IL-33 pre-treated (+IL33/+ConA) and untreated (-IL33/+ConA) mice lost weight in a similar manner during the first 24 hours after ConA injection (**Figure 7B**). However, IL-33 pre-treated mice started gaining weight within 2 days post-treatment and recovered more quickly than untreated mice. At the time of sacrifice, the texture of the livers from IL-33 pre-treated mice was slightly rough but otherwise healthy, while without IL-33 pre-treatment, the livers of the ConA injected mice appeared nodular and had discolored regions (**Supplementary Figure 7A**). Quantification of ILC2s by flow cytometry showed no differences in ILC2 numbers in -IL33/+ConA and +IL33/+ConA groups (**Figure 7C**). Intracellular TNFα and IFNɣ staining as well as cell surface staining of an activation marker CD69 showed that IL-33 pre-treatment had no effects on ConA-induced T cell activation at the time of analyses (**Supplementary Figure 7B**). Myeloid analyses also showed similar numbers of all populations analyzed, although neutrophils and macrophages appeared slightly higher in -IL33/+ConA group compared to +IL33/+ConA group (**Figure 7D**). Interestingly, IL-33 pre-treatment prevented increases in CD206^+^ macrophages upon ConA treatment (**Figure 7E**). Quantification of the transcripts of fibrosis-related genes by qPCR showed that *Col1a1, Acta2* and *Timp1* were more highly expressed in -IL33/+ConA livers compared to +IL33/+ConA treated livers (**Figure 7F**), indicating increased or delayed tissue-repair responses in -IL33/+ConA group compared to +IL33/+ConA group. Picrosirius red staining of type 1, 2 and 3 collagen fibers (48, 49) also indicated higher amounts of collagen fibers in -IL33/+ConA livers than +IL33/+ConA livers (**Figures 7G, H**).

To further investigate the role of ILC2s in ConA-induced hepatitis and fibrosis/cirrhosis, we examined CD127 cKO mice, which have very few ILC2s in the lung as well as liver. ConA treatment of CD127 cKO mice induced significantly less body weight loss compared to WT mice (**Figure 7I**) and IL-33 pre-treatment had no effect (**Figure 7J**). As expected, the ILC2 numbers of CD127 cKO mouse liver were about 10% or less of those in WT mice (**Figure 7K**). ConA treatment of CD127 cKO mice also induced no significant increase in neutrophils and CD206^+^ macrophages and IL-33 pre-treatment had little effect (**Figures 6L, M**). IL-33 pre-treatment also had no significant effect on the expression of fibrosis-related genes (**Figure 7N**) or the amounts of collagen fibers (**Figures 7O, P**). These results suggest that liver-resident ILC2s play an important role in ConA-induced hepatitis as previously reported (26), while lung-derived ILC2s exert protective effects.

It was previously shown that pre-treatment of mice with recombinant IL-6 protects from ConA-induced hepatic injury (50). To examine the effects of ILC2-derived IL-6 on ConA-induced injury, we i.v. injected ILC2s isolated from IL-33 treated WT or *Il6* ^-/-^ lungs (CD45.2^+^) into Pep3b mice (CD45.1^+^) and i.v. injected ConA two weeks later (**Supplementary Figure 7C**). Overall, there was no significant difference in the weight loss or fibrosis-associated gene expression between WT and *Il6* ^-/-^ ILC2 injected groups (**Supplementary Figures 7D, E**), indicating that ILC2-derived IL-6 is not responsible for the protective role of lung-derived ILC2s in the ConA model.

## Discussion

Our current study has shown that i.n. IL-33 administration induces local activation and proliferation of lung resident ILC2s. Although ILC2 numbers in PB, spleen and liver also increase following i.n. IL-33 administration, our parabiosis experiments showed that the increases in ILC2 numbers are due to migration of activated lung ILC2s to these tissues. While activated lung ILC2s circulate in PB, they preferentially migrate to the liver, and migration to other tissues, including the SI and BM, is negligible. Furthermore, the migration of activated lung ILC2s have significant effects on immunity and inflammation in the liver. Repeated i.n. IL-33 administration results in migration of high numbers of lung ILC2 to the liver leading to eosinophilic hepatitis and fibrosis-associated gene expression. On the other hand, pre-treatment of mice with i.n. IL-33 attenuates ConA-induced acute hepatitis and severe fibrosis/cirrhosis. These two apparently opposing effects of lung-derived ILC2s are likely mediated by cytokines produced by lung-derived ILC2s in the liver. Whereas the numbers of PB ILC2s return to naïve levels by 2 weeks after i.n. IL-33 administration, those in the liver remain high for more than 2 months. Moreover, lung-derived ILC2s in the liver continue to express intracellular IL-5 and IL-13 for more than two months after the i.n. IL-33 administration, while lung ILC2s quickly stop producing cytokines. This may be due to differential regulatory signals in the lung and liver environment or an intrinsic property of the subset that migrates to the liver. IL-5 produced by lung-derived ILC2s can drive eosinophilia while IL-13 can stimulate hepatic stellate cells and induce upregulation of fibrosis-associated genes (8) including *Col1a1*, *Acta2* and *Timp1*, thus promoting liver fibrosis. ILC2s are critical for the development of eosinophilic hepatitis and induction of fibrosis-related gene expression in the IL-33 treated mice as the ILC2-deficient CD127 cKO mice showed much reduced or no sign of eosinophilia and fibrosis. Although we have shown that IL-13 produced by lung-derived ILC2s also likely induces differentiation of macrophages into the M2 pathway and promotes type 2 immune environment in the liver, it is interesting to note, that IL-33 pre-treatment somehow prevented ConA-induced expression of CD206 in macrophages. It is possible that the signals involved in M2 differentiation in the ConA model may be different from those in the IL-33 model. In fact, there is no sign of ILC2 expansion upon ConA treatment. Therefore, it is unlikely that ILC2s actively produce IL-13 in +IL-33/+ConA livers. ConA-induced hepatitis is mediated, at least in part, by the prototypic Th1 cytokines IFNγ and TNFα (51). As the IL-33 pre-treatment has no obvious effects on T cells expressing intracellular TNFα or IFNγ (**Supplementary Figure 7B**), it is also unlikely that lung-derived ILC2s inhibit T cell activation by ConA. Instead, priming of the liver environment towards the type 2 immunity driven by lung-derived ILC2s likely favors repair of damaged liver tissues as suggested by the rapid recovery from the initial weight loss in IL-33 pre-treated mice. Lung-derived liver ILC2s also produce IL-6, a hepatocyte growth factor (52, 53), which is known to protect mice from ConA-induced liver injury (50), but IL-6 deficiency in ILC2s had no effect on the recovery from ConA-induced liver damage and consequently, the role of ILC2-derived IL-6 remains unknown.

Although the exact mechanisms by which activated lung ILC2s migrate out of the lung, circulate through PB and settle in the liver are still under investigation, we hypothesized that CD103 and CXCR6 may play a role. The αEβ7 integrin, formed by the pairing of CD103 with the β7 chain, binds E-cadherin on epithelial cells (44), and CD103 KO mice have greatly reduced numbers of intraepithelial lymphocytes (54). Because the majority of activated lung ILC2s express CD103 whereas ILC2s in PB and liver in the same mice are CD103^-^, it seemed likely that the activated lung ILC2s expressing CD103 may be retained in the lung by the interaction between CD103 and E-cadherin, while CD103^-^ ILC2s migrate out of the lung, circulate through PB and settle in the liver. I.n. administration of IL-33 into WT and *Itgb7* ^-/-^ mice led to higher numbers of ILC2s in the PB of *Itgb7* ^-/-^ mice compared to WT mice, in line with the hypothesis. However, ILC2s were also increased in the lungs of *Itgb7* ^-/-^ mice compared to WT mice. This suggested that the elevated ILC2s in the PB of *Itgb7* ^-/-^ are most likely due to increased ILC2 expansion in the lungs of *Itgb7* ^-/-^ mice. Therefore, CD103 seems to negatively regulate lung ILC2 activation/proliferation but not their emigration.

As most ILC2s in PB and liver express the chemokine receptor CXCR6, which mediates migration of NKT cells into the liver (43), we also investigated the effects of the absence of CXCR6 on ILC2 migration using *Cxcr6* ^gfp/gfp^ mice. It was previously reported that naïve and papain treated *Rag* ^-/-^ *Cxcr6* ^gfp/gfp^ mice have reduced ILC2s compared to *Rag* ^-/-^ mice (45). To avoid complications due to the developmental defect in ILC2s in *Cxcr6* ^gfp/gfp^ mice, we investigated ILC2 migration by transplanting IL-33 activated ILC2s from the lungs of WT and *Cxcr6* ^gfp/gfp^ mice. Although we found slightly less transplanted ILC2s in the livers of the mice that received *Cxcr6* ^gfp/gfp^ lung ILC2s than those that received WT lung ILC2s, the differences were not statistically significant. Thus, while we cannot exclude the possibility that CXCR6 is involved in lung ILC2 migration to the liver, it is not necessary for their migration, at least early on. On the contrary, FTY720 mediated inhibition of S1PR signaling revealed that lung ILC2 circulation and migration to the liver are partially dependent on S1PR. Further investigation involving transcriptomic and proteomic approaches and genetically modified mouse models is required to identify the specific molecules involved in emigration of ILC2s from the lung and their migration into the liver.

Since the emergence of the idea of tissue resident ILCs (28, 29), multiple reports have shown the ability of ILC2s to traffic between different organs. Stier et al. reported migration of ILC2 progenitors out of the BM upon systemic IL-33 injections, while Karta et al. demonstrated β2 integrin mediated recruitment of ILC2s from the BM to the lung upon allergen stimulation (30, 31). A report by Huang et al. also demonstrated S1P-dependent trafficking of inflammatory ILC2s from the intestine to the lung (33). More recently, Ricardo-Gonzalez et al. reported circulation of intestinal and lung ILC2s after helminth infections, using fate-mapping methods (34). Our parabiosis results agree with the reports by Gasteiger et al. in terms of expansion of the tissue resident ILC2s upon i.n. IL-33 treatment. The discrepancy between our results and reports by Karta et al. is likely due to differences in the ways ILC2s are defined. Karta et al. defined ILC2s as Lin^-^Thy1^+^. In our hands, about 50% of Lin^-^Thy1^+^ lung lymphocytes are GATA3^-^ non-ILC2s and are not tissue resident as they are mixtures of CD45.1^+^ and CD45.2^+^ cells in our parabiotic mice. In our studies, ILC2s are defined by Lin^-^Thy1^+^CD127^+^ST2^+^, and they are all GATA3^+^. Our parabiosis results also showed that i.n. IL-33 administration has little effect on BM ILC2s and does not induce recruitment of ILC2s from other tissues to the lung.

Asthma patients have been reported to have high numbers of ILC2s in PB (23), but their origins are unknown. Our current study suggests that PB ILC2s in asthma patients are likely derived from the patients’ allergen-stimulated lungs. It still remains to be determined whether PB ILC2s in asthma patients also settle in the liver and regulate hepatic inflammation and fibrosis. There have been case reports of patients simultaneously suffering from pulmonary fibrosis and liver cirrhosis (55) as well as asthmatic patients with eosinophilic hepatitis or cirrhosis (56, 57). However, our current study has shown that lung-derived liver ILC2s can also be protective against ConA-induced acute hepatitis. Understanding the precise roles of lung-derived ILC2s would require detailed immune cell profiling, histological and transcriptomic analyses of liver biopsy samples from asthmatic patients showing signs of hepatitis, liver fibrosis or cirrhosis. It is important to note that the data presented here are contradictory to a previously published study demonstrating a pro-inflammatory role of liver ILC2s in the ConA-induced hepatitis model (26). This may be due to functional differences between lung-derived ILC2s and liver resident ILC2s. In our hands, i.n. IL-33 pre-treatment provided protective effects from ConA-induced damage. In contrast, ILC2 depletion ameliorated ConA-induced hepatitis, whereas adoptive transfer of hepatic ILC2s isolated from i.p. IL-33 treated mice aggravated the disease in the studies by Neumann et al. (26). In CD127 cKO mice, which have significantly reduced numbers of lung-derived and liver resident ILC2s, we have shown that IL-33 pre-treatment had little effect in promoting recovery, but ConA treatment also did not induce severe sickness in the first place. These results suggest that lung-derived ILC2s are protective, while liver resident ILC2s are pathological for ConA-induced hepatitis. Further studies will be required to delineate the mechanisms by which the lung-derived and liver resident ILC2s exert opposing roles in the ConA hepatitis model. If confirmed and extended, these new findings could pave the way for improved therapeutic intervention in the treatment of hepatitis patients.

## Data Availability Statement

The original contributions presented in the study are included in the article/**Supplementary Material**. Further inquiries can be directed to the corresponding author.

## Ethics Statement

All animal use was approved by the animal care committee of the University of British Columbia in accordance with the guidelines of the Canadian Council on Animal Care.

## Author Contributions

Conceptualization: LM and FT. Methodology: LM and FT. Investigation: LM, MR-H, CS, YY, MO, HS, and CC. Validation: LM. Visualization: LM and FT. Funding acquisition: FR and FT. Supervision: FR and FT. Resources: CC, FR, and FT. Writing – original draft: LM and FT. Writing – review & editing: LM, MR-H, CS, YY, MO, HS, FR, and FT. All authors contributed to the article and approved the submitted version.

## Funding

LM is a recipient of a UBC Four Year Fellowship and a Vanier Canada Graduate Scholarship. CS is a recipient of a UBC Four Year Fellowship and a Canadian Institute of Health Research (CIHR) studentship and post-doctoral fellowship. This work was supported by grants from the CIHR (PJT-153304, MOP-126194, FDN-159908) and the Heart and Stroke Foundation of Canada (G-19-0026541).

## Conflict of Interest

The authors declare that the research was conducted in the absence of any commercial or financial relationships that could be construed as a potential conflict of interest.
