# Supplementary material for: Migration of Lung Resident Group 2 Innate Lymphoid Cells Link Allergic Lung Inflammation and Liver Immunity
Source: Front Immunol. 2021 Jul 9;12:679509. doi: 10.3389/fimmu.2021.679509 (PMC8299566; doi:10.3389/fimmu.2021.679509)
Supplement: Supplementary file 1 [file Image_1.pdf]

Supplementary Material

Supplementary Figures

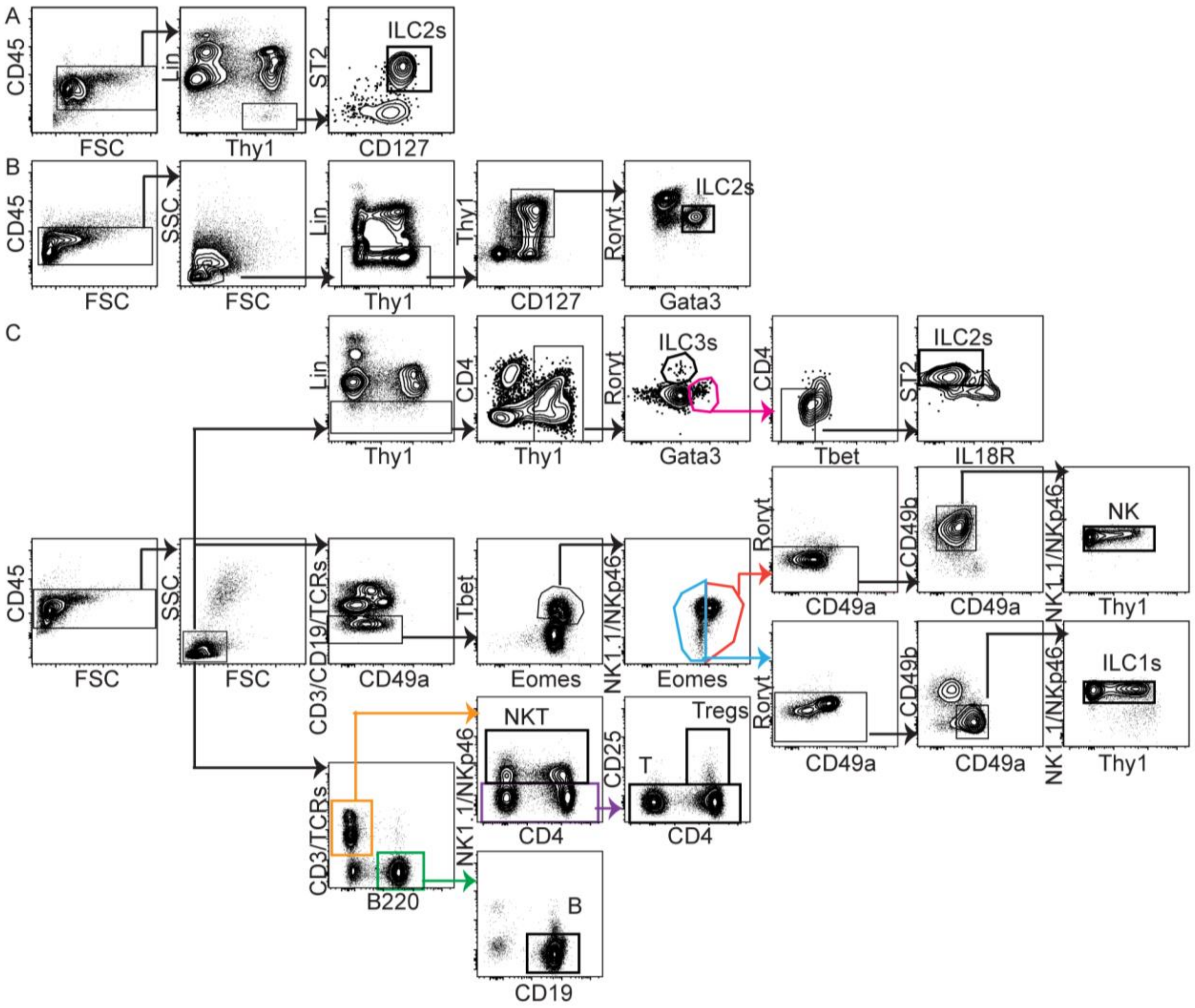

Supplementary Figure 1. Gating strategies used to identify various lymphocyte populations

Gating strategies used to identify ILC2s in the lung, liver, PB, spleen, mediastinal LN and BM based on surface markers **(A)**, ILC2s in small intestine and mesenteric lymph node **(B)**, and various lymphocyte populations in the lung and liver **(C)**. Lineage cocktail in A and B contains CD3ε, CD4, CD19, TCRβ, TCRγδ, CD11b, CD11c, NK1.1, Ter119 and Gr-1, and that in C is the same as A and B without CD4. NK=natural killer cells, NKT=natural killer T cells, Tregs=regulatory T cells.

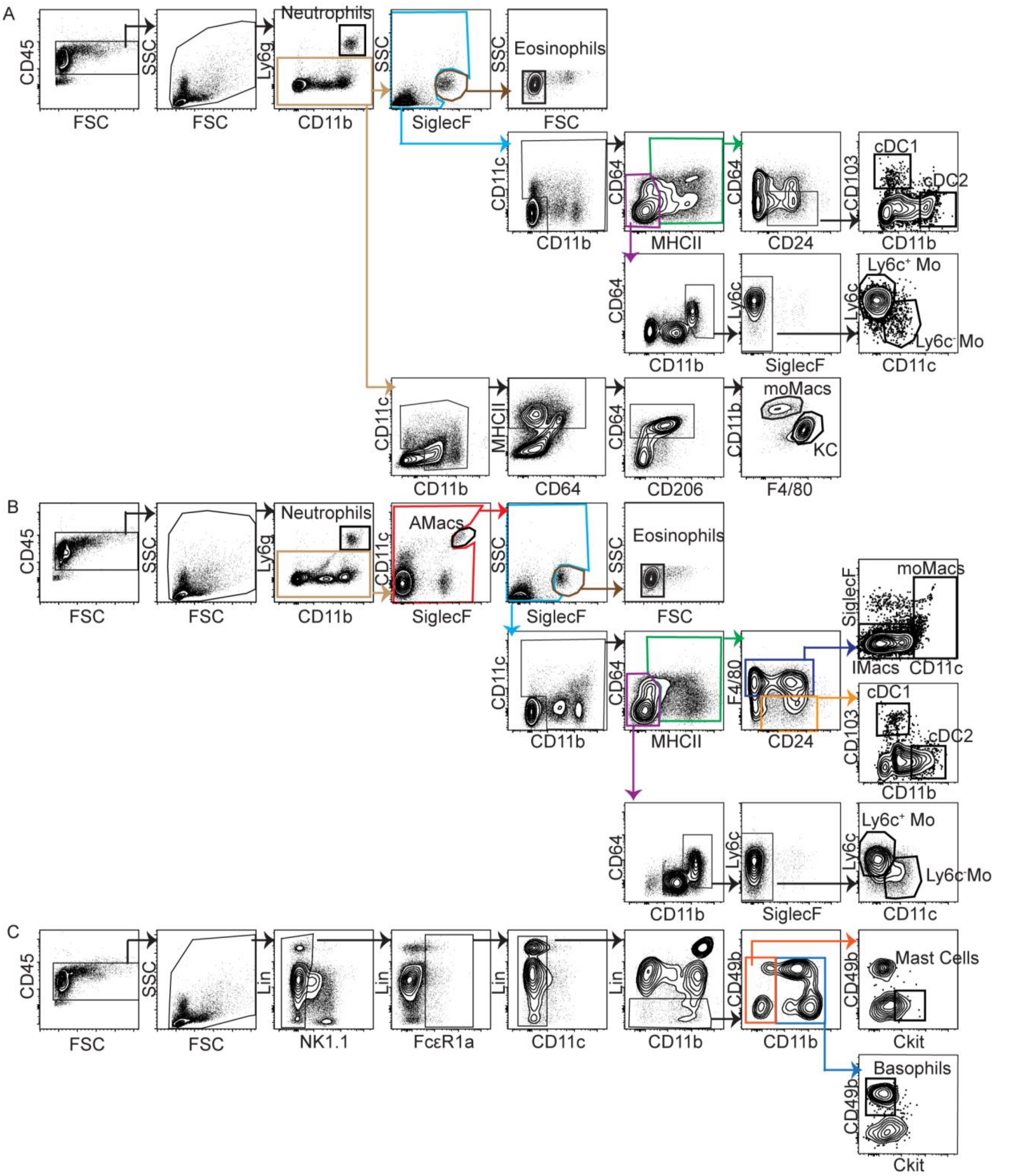

Supplementary Figure 2. Gating strategies used to identify various myeloid populations

Gating strategies used to identify various myeloid populations in the liver **(A)** and lung **(B),** and basophils and mast cells in the liver and lung **(C)**. Lineage cocktail in C contains CD3ε, CD5, CD19, Ter119 and Gr-1. cDC1= type 1 conventional dendritic cells, cDC2= type 2 conventional dendritic cells, Mo=monocytes, KC=Kupffer cells, moMacs=monocyte-derived macrophages, AMacs=alveolar macrophages, IMacs=interstitial macrophages.

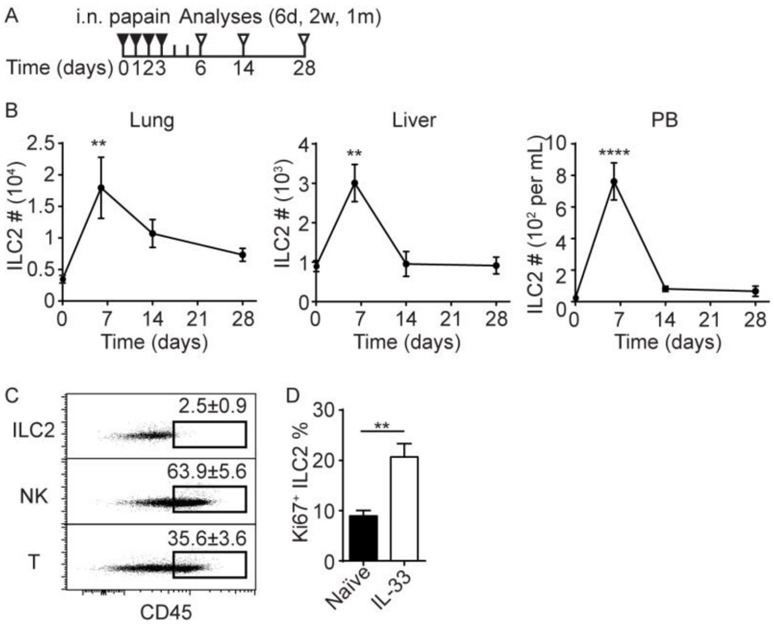

Supplementary Figure 3. ILC2s undergo expansion and contraction in the lung, liver and PB upon papain treatment

(**A**) Mice received four daily i.n injections of papain and were analyzed at various time points. d=day, w=week, m=month. (**B**) ILC2s were quantified in the lung, liver and PB after i.n papain administrations. Day 0 is naïve. Asterisks indicate significant differences from naïve. **(C)** Mice were in vivo labeled with CD45 antibody and the percentages of $CD45^+$ ILC2s, NK cells and T cells were analyzed in the liver. **(D)** The percentage of $Ki67^+$ ILC2s was quantified in the BM before and 3 days after IL-33 treatment (day 5 in Figure 1C). n=5-10; ≥2 independent experiments (B), n=5; 2 independent experiments (C), n=5-8; 2 independent experiments (D). Data shown are mean ± SEM. One-way ANOVA with Bonferroni correction (B) or unpaired two-tailed t test (D) was used to determine statistical significance. **P≤0.01, ****P≤0.0001.

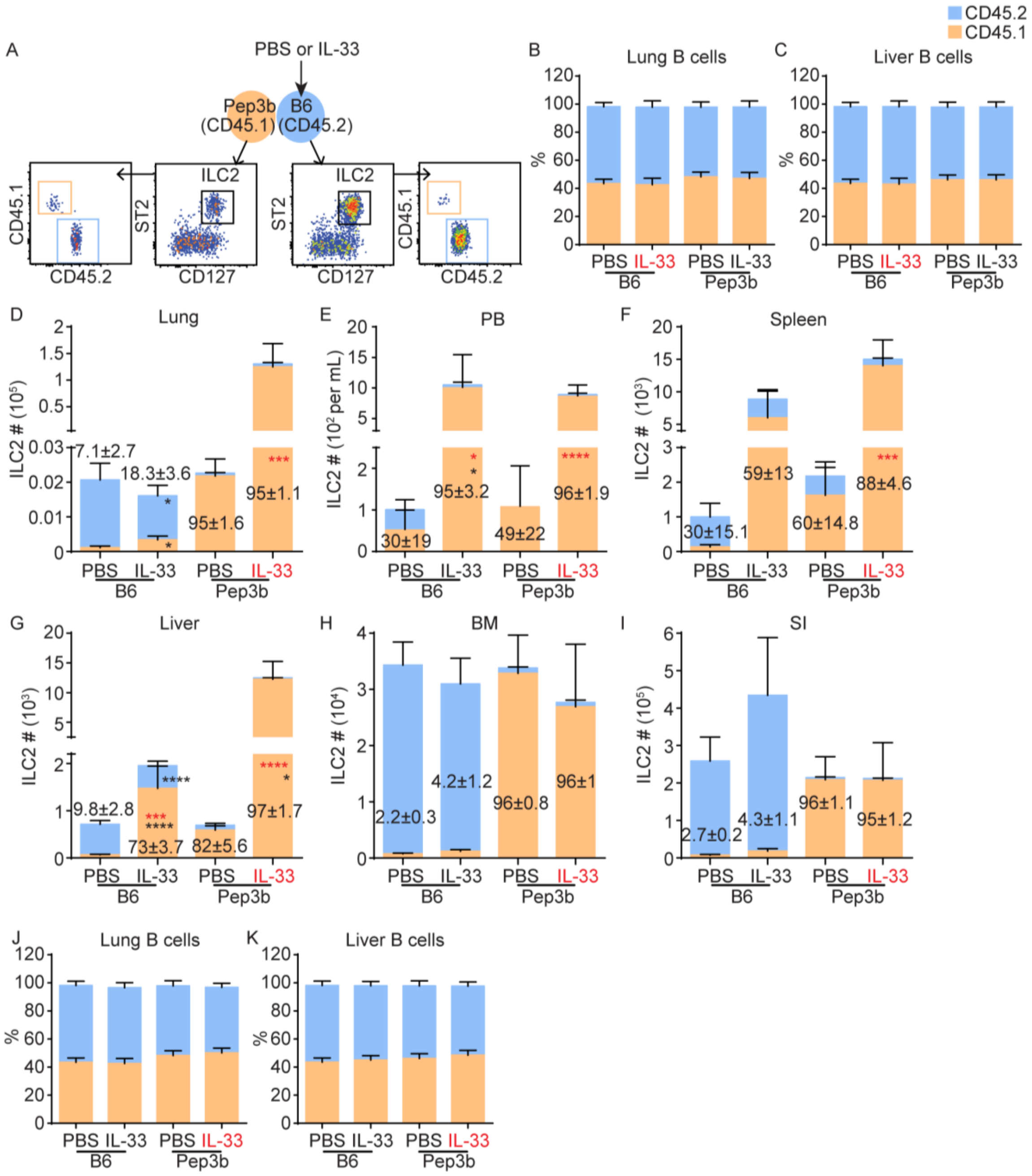

Supplementary Figure 4. ILC2s migrate from the lung to the liver after i.n. IL-33 injections into Pep3b mice

(**A**) B6 and Pep3b mice were conjoined by parabiosis surgery. B6 (data shown in Figure 2, Supplementary Figures 4B and C) or Pep3b (data shown in Supplementary Figures 4D-K) mice were given three daily i.n. PBS or IL-33 injections and both B6 and Pep3b mice were analyzed on day 7 (see Figure 1C). The schematic shown here represents i.n. injections into B6 mice. (**B and C**) Percentages of CD45.1^{+} (orange) and CD45.2^{+} (blue) B cells in the lung (B) and liver (C) of mice indicated on x-axes after i.n. PBS/IL-33 administration into B6 mice. (**D-I**) Numbers and percentages of CD45.1^{+} (orange) and CD45.2^{+} (blue) ILC2s in the lung (D), PB (E), spleen (F), liver (G), BM (H) and SI (I) after i.n. PBS/IL-33 injections into Pep3b mice. X-axes show the strains of mice analyzed

(B6 or Pep3b) and treatment groups (PBS or IL-33). IL-33 highlighted in red indicates the mouse that received injections. Numbers within graphs indicate the percentages of $CD45.1^+$ ILC2s. Black and red asterisks indicate statistical significance of percentages and cell numbers, respectively, of $CD45.2^+$ (within blue bars) and $CD45.1^+$ (within orange bars) ILC2s compared to PBS treated pairs. (**J and K**) Percentages of $CD45.1^+$ (orange) and $CD45.2^+$ (blue) B cells in the lung (J) and liver (K) of mice indicated on x-axes after i.n. PBS/IL-33 administration into Pep3b mice. Data shown are mean ± SEM. n=6-7; ≥3 independent experiments (B and C) or n=4-6 (except SI, where n=3); ≥3 independent experiments (D-K). Two-way ANOVA with Bonferroni correction was used to determine statistical significance. *P≤0.05, ***P≤0.001, ****P≤0.0001.

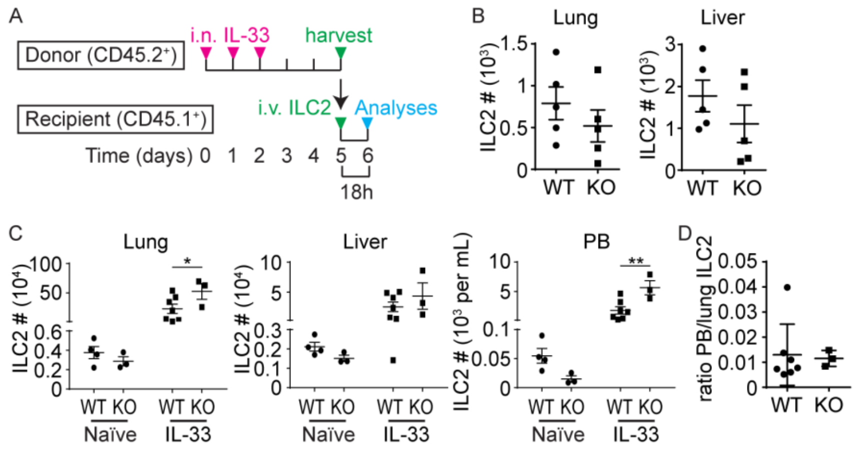

Supplementary Figure 5. CD103 and CXCR6 are not critical for lung ILC2 migration to the liver

(**A**) ILC2s were isolated from IL-33 treated WT or *Cxcr6*$^{gfp/gfp}$ (KO) lungs (donor) and intravenously injected into Pep3b mice (recipient). The recipients' lungs and livers were analyzed 18 hours after the injection. (**B**) Donor ($CD45.2^+$) ILC2s were quantified in the lungs and livers of the recipient mice. (**C**) WT and *Itgb7*$^{-/-}$ (KO) mice were treated with three daily i.n. injections of IL-33. ILC2s were quantified in the lung, liver and PB of naïve (circle) and treated (square) mice 3 days after the last injections (day 5 in Figure 1C). (**D**) The ratio of PB and lung ILC2s after IL-33 treatment was determined in WT and *Itgb7*$^{-/-}$ (KO) mice. Data shown are mean ± SEM. n=5; 2 independent experiments (B) or n=3-7; 1 (*Itgb7* KO) or 2 (WT) independent experiments (C and D). Unpaired two-tailed t test (B and D) or two-way ANOVA with Bonferroni correction (C) was used to determine statistical significance. *P≤0.05, **P≤0.01.

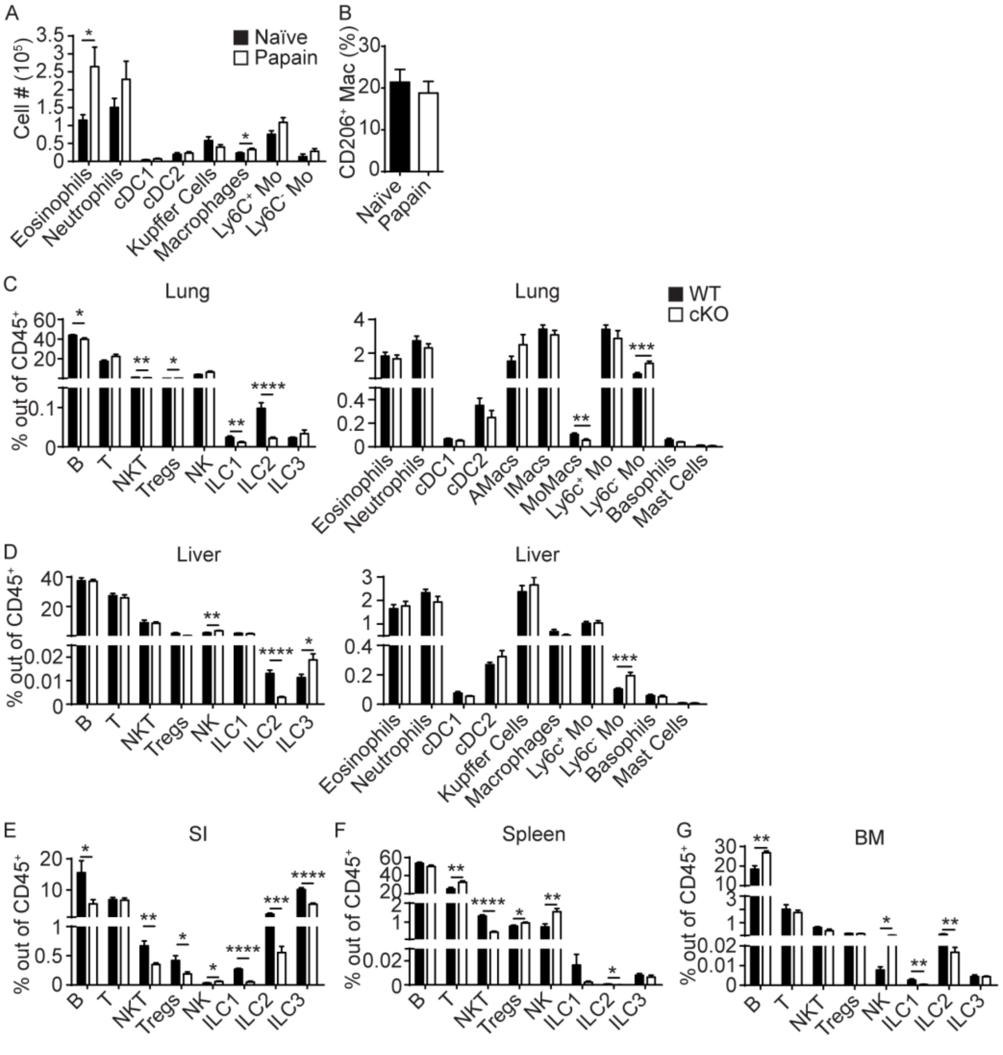

Supplementary Figure 6. CD127 cKO mice are deficient in ILC2s

(**A and B**) Mice received four daily i.n papain injections and the livers were analyzed for various myeloid populations (A) and $CD206^{+}$ macrophages (B) on day 6 (see Figure 1A). Black bars=naïve, white bars=papain treated. (**C-G**) Percentages of various lymphocyte populations out of $CD45^{+}$ cells were quantified in the lung (C, left), liver (D, left), SI (E), spleen (F) and BM (G), and myeloid populations were quantified in the lung (C, right) and liver (D, right) of naïve WT (black bars) and CD127 cKO (white bars) mice. Tregs=regulatory T cells, cDC1=conventional type 1 DCs ($CD103^{+}$), cDC2=conventional type 2 DCs ($CD11b^{+}$), Mo=monocytes. Data shown are mean ± SEM. n=5-6; ≥2 independent experiments (A and B), n=5-18; ≥2 independent experiments (C-G). Unpaired two-tailed t test was used to determine statistical significance. *P≤0.05, **P≤0.01, ***P≤0.001, ****P≤0.0001.

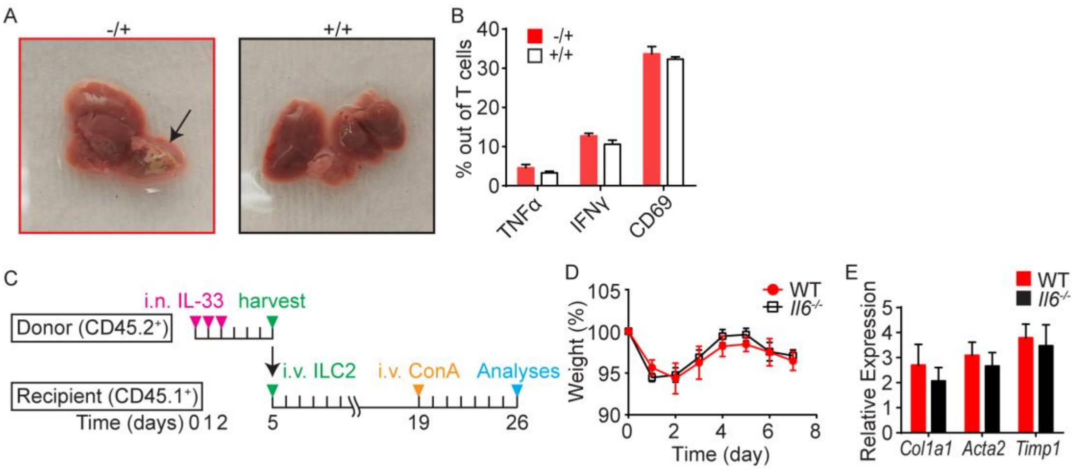

Supplementary Figure 7. ILC2-derived IL-6 is not required for tissue repair upon ConA treatment

(**A and B**) WT mice were treated and analyzed as described in Figure 7A. (A) Pictures of livers collected from -IL33/+ConA (left) and +IL33/+ConA (right) mice. The arrow indicates discolored region. (B) Percentages of T cells positive for indicated markers. Red=-IL33/+ConA, white=+IL33/+ConA. (**C-E**) ILC2s isolated from WT or *Il6*$^{-/-}$ (CD45.2^{+}) lungs on day 5 after i.n. IL-33 administration were i.v. injected into Pep3b mice (CD45.1^{+}). ILC2 transplanted Pep3b mice were treated with i.v. injections of ConA 2 weeks later and analyzed 1 week after the ConA treatment (C). (D and E) Mice transplanted with WT (red) or *Il6*$^{-/-}$ (black) lung ILC2s were monitored for body weight loss after ConA injections (D) and relative expression of indicated genes was analyzed in the liver (E). Data shown are mean ± SEM. n=4; 2 independent experiments (A and B), or n=6; 2 independent experiments (D and E). Unpaired two-tailed t test was used to determine statistical significance.
